# The Follicular Immune Checkpoint: PD-1/PD-L1 and Immune Tolerance in Oocyte Competence and IVF Failure

**DOI:** 10.3390/ijms27135712

**Published:** 2026-06-24

**Authors:** Charalampos Voros, Chrysi Christodoulaki, Ioanna Petrakou, Rafaela Panagopoulou, Ioanna Zouganeli, Dimos Sioutis, Fotios Chatzinikolaou, Georgios Papadimas, Georgios Daskalakis, Periklis Panagopoulos

**Affiliations:** 1 Department of Obstetrics and Gynecology, ‘Alexandra’ General Hospital, National and Kapodistrian University of Athens, 80 Vasilissis Sofias Avenue, 11528 Athens, Greece; gdaskalakis@yahoo.com; 2 Department of Obstetrics and Gynecology, General Hospital of Chania, 73300 Chania, Greece; christodoulakichr@hotmail.com; 3 Third Department of Obstetrics and Gynecology, Attiko University Hospital, Medical School, National and Kapodistrian University of Athens, 12462 Athens, Greece; petrakouioanna@hotmail.com (I.P.); rafaelapng@hotmail.com (R.P.); joannazouga97@gmail.com (I.Z.); dsioutis@gmail.com (D.S.); paninosrafaela@yahoo.gr (P.P.); 4 MSc Program in High-Risk Pregnancy, School of Medicine, National and Kapodistrian University of Athens, 16673 Athens, Greece; 5 Laboratory of Forensic Medicine and Toxicology, School of Medicine, Aristotle University of Thessaloniki, 54124 Athens, Greece; fotischatzin@auth.gr; 6 Athens Medical School, National and Kapodistrian University of Athens, 15772 Athens, Greece; dr.georgepapadimas@gmail.com

**Keywords:** PD-1, PD-L1, follicular fluid, granulosa cells, immune checkpoint, oocyte competence, ovarian follicle, extracellular vesicles, cytokines, immune tolerance, polycystic ovary syndrome, endometriosis, implantation failure, reproductive immunology

## Abstract

Oocyte formation occurs successfully within a meticulously controlled follicular environment characterized by well-documented endocrine, metabolic, and paracrine signals. Yet, the immunological landscape of the follicle and its role in influencing oocyte competency has received less attention in research. Growing research indicates that the ovarian follicle functions as an immunological-active niche necessitating a precise equilibrium between controlled inflammation and targeted immune tolerance. The programmed cell death-1 (PD-1) receptor and its ligand PD-L1 constitute a crucial immune checkpoint pathway, essential for sustaining peripheral immunological tolerance and averting excessive immune activation. Despite their comprehensive research in cancer biology and maternal–fetal interactions, their possible function in the follicular microenvironment remains mostly unexamined. We propose that PD-1/PD-L1 signaling may facilitate the formation of a localized immune-tolerant milieu inside the follicle to safeguard the developing oocyte from inflammatory injury and immune-mediated stress. The disturbance of this suggested equilibrium may lead to a pro-inflammatory follicular environment, compromised granulosa cell function, and modified oocyte maturation, hence affecting fertilization and embryonic developmental potential. In clinical contexts with immunological dysregulation, such as endometriosis, polycystic ovarian syndrome, and unexplained IVF failure, such processes may be especially significant. The purpose of this narrative review is to assimilate the current comprehension of immune regulation in the follicle with the established biology of PD-1/PD-L1 and to investigate a potential correlation between immune checkpoint signaling, oocyte competence, and assisted reproductive outcomes. Considering the follicle as an immune-regulated microenvironment offers a new paradigm for comprehending infertility and identifying novel indicators or therapeutic targets.

## 1. Introduction

Oocyte formation transpires inside a meticulously regulated follicular niche, shaped by endocrine, metabolic, and paracrine signals. Traditional models of folliculogenesis have concentrated on gonadotropin-mediated pathways and the interaction between granulosa cells and oocytes, with hormonal control as the focal point of oocyte competence [1]. Nevertheless, mounting data indicates a more intricate milieu whereby localized immunological activity plays a role in follicular homeostasis and reproductive capacity.

Immune cells are not only temporary or incidental inside the preovulatory follicle. Macrophages, T lymphocytes, dendritic cells, and other immune groups are constantly detected in follicular fluid, often at ratios equivalent to granulosa cells [2]. Changes in immune-cell composition have been documented in many clinical contexts, including inadequate ovarian response, polycystic ovary syndrome, endometriosis, and obesity. Alterations in T-cell populations, macrophage polarization, and cytokine expression have been linked to diminished oocyte quality, fertilization, and embryo development, indicating that immunological dysregulation may directly affect reproduction [3].

Follicular fluid is a dynamic signaling environment rather than a passive ultrafiltrate. Cytokines, chemokines, growth factors, and metabolic intermediates constitute a closely interconnected network capable of modulating granulosa cell activity and oocyte maturation [4]. Data from both natural and induced cycles indicate that variations in this microenvironment are associated with follicular development, steroidogenesis, and embryo viability. In pathological circumstances like endometriosis or metabolic dysregulation, this equilibrium seems to transition to a pro-inflammatory state, often linked to alterations in the gene expression of granulosa cells and diminished responsiveness to gonadotropins [5].

A significant conceptual challenge in follicular biology is the coexistence of physiological inflammatory activity and local immunological tolerance within the same milieu. Ovulation mimics a temporary inflammatory process characterised by cytokine release, immune cell recruitment, tissue remodeling, and vascular alterations. A hyper-inflammatory condition or prolonged inflammatory activation might adversely affect granulosa cell function and oocyte developmental competence. Consequently, the follicular milieu presumably necessitates precisely regulated systems to provide controlled inflammatory signaling while mitigating excessive immune-mediated damage. Immune-checkpoint pathways have a role in several physiological systems by diminishing T-cell activation and preventing tissue damage. Comparable regulatory mechanisms may be implicated in sustaining local immunological homeostasis throughout the periovulatory phase inside the ovarian follicle [6].

Programmed cell death protein-1 (PD-1) and its ligands, PD-L1 and PD-L2, have been well described in cancer biology and the formation of fetomaternal tolerance. Their function in the ovarian follicle has just lately begun to surface. PD-1 and its ligands have been shown to be present in human ovarian tissue, including granulosa cells, oocytes, and follicular fluid collected during assisted reproduction [7]. Experimental data suggest that follicular fluid may influence T-cell activity via PD-1-related pathways, hence endorsing the potential for local checkpoint-mediated immune control inside the follicular compartment. Our findings suggest that PD-L1 is expressed on extracellular vesicles (EVs) produced from follicular fluid, suggesting a possible way for immune regulation to occur inside the follicular environment via localized vesicle mediation [8].

Concurrent studies in granulosa cells suggest a possible mechanistic connection between immunological signaling and follicular integrity. Activation of PD-L1-related pathways has been associated with the decrease in granulosa cell apoptosis via PI3K/AKT signaling, particularly in pathological circumstances like polycystic ovarian syndrome [9]. Cytokine-mediated alterations, particularly via interferon-γ and chemokine signaling, impair granulosa cell function by downregulating essential genes associated with steroidogenesis and follicular response. Taken together, our results point to the possibility that granulosa cell survival and oocyte competence under immune-related stress are impacted by PD-1/PD-L1-associated signalling. Nonetheless, direct functional evidence in vivo is still limited [10].

Recent evidence suggests that immune regulation may play a broader role in follicular biology and oocyte maturation than previously appreciated. However, the precise contribution of PD-1/PD-L1 signaling within this network remains unclear. An in-depth examination of immune checkpoint activity inside the follicular milieu may provide new insights into unexplained infertility, suboptimal ovarian response, and the inconsistent results seen in assisted reproduction. Current evidence on immune activity in follicular fluid is summarized in this narrative review, with a particular emphasis on granulosa cell function, PD-1/PD-L1 signaling, and cytokine-mediated regulation. This study integrates genetic, cellular, and clinical data to investigate the notion that a follicular immunological checkpoint exists and may influence oocyte competency and IVF results.

## 2. Materials and Methods

The existing evidence regarding immune regulation in the follicular microenvironment has been identified and synthesized through a structured narrative approach, focusing on PD-1/PD-L1 signaling, follicular fluid composition, granulosa cell biology, and assisted reproduction outcomes. The relevant papers were sourced from PubMed/MEDLINE and PubMed Central, limited to publications published in English, including both experimental and clinical research. The search criteria were chosen to include the convergence of reproductive biology and immunology. The keywords included “PD-1,” “PD-L1,” “ovary,” “follicular fluid,” “granulosa cells,” “immune cells,” “cytokines,” “IVF,” “PCOS,” “endometriosis,” and “poor ovarian response.” Additional combinations included “extracellular vesicles,” “immune checkpoint,” “T cells,” and “oocyte competence” for investigations evaluating both molecular pathways and clinical correlations. Key papers were examined in reference lists to ensure the inclusion of pertinent research that may not have been identified via database searches alone.

Studies were chosen for their pertinence to the primary hypothesis of immune regulation inside the follicle. Research prioritizing human follicular fluid, ovarian tissue, or granulosa cells was emphasized, particularly studies addressing reproductive outcomes like oocyte quality, fertilization rates, embryo development, or implantation. Experimental research on immunological checkpoint signaling, T-cell modulation, cytokine-mediated effects, and intracellular pathways such as PI3K/AKT were included where they offered mechanistic insights into follicular physiology. The selective use of reviews was implemented to bolster the biological background, particularly in instances when primary human data is scarce.

While the data was not rigorously evaluated according to systematic review standards, it was topically structured, thereby synthesizing results across molecular, cellular, and clinical dimensions. The focus was on identifying convergent patterns, including alterations in immune-cell composition within follicular fluid, variations in cytokine profiles under pathological conditions, and growing evidence of checkpoint-mediated immune regulation. Particular emphasis was placed on research demonstrating functional linkages among immunological signals, granulosa cell survival, steroidogenesis, and oocyte competence.

A quantitative synthesis was considered unsuitable because of the variability in research designs, patient demographics, and laboratory techniques. The results were analyzed concerning differences in stimulation procedures, follicular sample techniques, and analytical methodologies. Conclusions were made judiciously, grounded on biological plausibility and consistency across separate observations rather than statistical aggregation. By emphasizing potential functions for PD-1/PD-L1 signaling as a local regulatory mechanism, the current narrative synthesis seeks to provide a cohesive framework connecting immune activity in the follicular environment and oocyte maturation.

The analysis of the presently available evidence is significantly obstructed by considerable methodological heterogeneity among studies, encompassing variations in ovarian stimulation protocols, follicular fluid collection techniques, extracellular vesicle isolation methods, patient selection criteria, and laboratory analytical platforms.

## 3. The Follicular Microenvironment as an Immune-Regulated Niche

### 3.1. Cellular Composition of the Follicular Immune Compartment

Follicular architecture incorporates immune-cell populations that are geographically and functionally integrated inside the granulosa–oocyte complex, rather than being scattered peripherally. Examination of preovulatory follicles indicates that immune cells are not confined to vascular compartments but are also found within the follicular fluid aspirated alongside granulosa cells, suggesting that the oocyte is directly subjected to immune-derived signals [11]. A significant segment of this population consists of macrophages exhibiting phenotypic plasticity that seems to be closely linked to local signaling gradients. The expression profiles of inducible nitric oxide synthase, arginase-1, and scavenger receptors indicate that macrophages in the follicle do not adhere rigidly to standard M1/M2 classifications but rather exist in intermediate activation states shaped by cytokine and metabolic signals [12].

The behaviour of macrophages dynamically alters and affects follicular expansion and ovulatory preparedness via the production of matrix metalloproteinases and tissue inhibitors that remodel the extracellular matrix [13]. In conjunction with the concurrent release of vascular endothelial growth factor and angiopoietins, macrophage activity is also linked to angiogenesis, the mechanism of nutrition delivery and hormonal exchange. Interruption of these channels may modify the vascular permeability of the follicle, thereby affecting the composition of the follicular fluid and the exposure of granulosa cells to systemic stimuli [14].

The populations of T-cell follicles are defined by antigen-experienced lymphocytes instead of naïve lymphocytes. The heightened expression of activation markers and cytokine production capability indicates previous priming, suggesting that follicular immune responses are influenced by both systemic immunological history and local cues [15]. Alterations in CD4^+^ and CD8^+^ subsets influence cytokine profiles, mostly via interferon-γ-driven pathways that regulate cellular metabolism and death. Regulatory T cells, which are not well defined in follicular research, likely contribute to the modulation of excessive immunological activation. In ovarian insufficiency models, data indicates that a deficiency in Tregs induces Th1-mediated responses, culminating in granulosa cell death via interferon-γ and tumour necrosis factor-α signaling [16].

Dendritic cells inside the follicle participate in antigen presentation, with cDC2 subsets being more prevalent in scenarios of compromised ovarian response. The expression of costimulatory markers and cytokine secretion patterns indicates that these cells may influence local T-cell development, either augmenting or inhibiting immunological activation based on the environment [17]. Despite their temporary nature, neutrophils provide proteolytic enzymes and reactive oxygen species that facilitate tissue remodeling associated with ovulation, therefore connecting innate immune activity with structural alterations in follicles. The main immune cell populations within the follicular microenvironment and their functional roles are summarized in Table 1.

### 3.2. Cytokine Signaling and Molecular Crosstalk

Exposure to cytokines triggers intracellular signalling pathways that directly influence steroidogenesis and cell viability in granulosa cells. The binding of interferon-γ to its receptor stimulates the JAK/STAT pathway, hence augmenting the transcription of genes related to immune response and apoptosis [18]. Co-exposure to tumour necrosis factor-α stimulates NF-κB signalling. Additionally, this improves the expression of inflammatory genes and further increases the sensitivity of cells to apoptotic stimuli. The interaction between these pathways enhances downstream effects, including the inhibition of essential regulators of follicular activity, such as the FSH receptor and aromatase. Decreased FSHR expression leads to lessened gonadotropin stimulation, whereas reduced CYP19A1 activity results in impaired oestradiol production, hence modifying the hormonal milieu essential for oocyte maturation [19].

Granulosa cells’ survival is significantly influenced by the PI3K/AKT signalling pathway, which appears to be regulated by immune-derived signals. The activation of this pathway results in enhanced cell proliferation, suppression of apoptosis via downstream targets like BCL-2, and facilitation of the metabolic activities necessary for follicular expansion [20]. Inflammatory circumstances affect PI3K/AKT signalling, shifting the balance towards pro-apoptotic pathways, resulting in heightened caspase activation and mitochondrial dysfunction. Reactive oxygen species produced during inflammatory signalling contribute to cellular stress, impairing mitochondrial DNA and obstructing ATP synthesis [21].

The modulatory effects of anti-inflammatory cytokines and type 2 immune mediators partially counterbalance pro-inflammatory signalling. IL-4 and IL-13 may alter macrophage polarisation and suppress the production of inflammatory mediators. However, their presence does not inherently restore complete immunological homeostasis in the context of persistent inflammatory stimulation [22]. Divergences in systemic and follicular cytokine profiles indicate that local regulatory systems function autonomously, and that follicular fluid has a distinct immunological identity, even within systemic inflammation. Metabolic variables intersect with cytokine signalling at several levels [23]. Lipid buildup, impaired glucose metabolism, and oxidative stress influence immune cell function and cytokine production, resulting in positive feedback loops that sustain inflammatory signalling. In metabolic illnesses, immunological activation and metabolic dysregulation are mutually reinforcing, which results in lasting abnormalities in granulosa cell function [24]. Such relationships are especially noticeable in metabolic disorders. Key cytokines and their associated intracellular signaling pathways in granulosa cells are outlined in Table 2.

### 3.3. Immune Imbalance in Pathological Follicular Environments

Common mechanisms are observed at a level of granulosa cell dysfunction and reduced oocyte competence. In polycystic ovarian syndrome, localised modifications persist in the follicle despite hormonal stimulation, accompanied by systemic inflammation. An adaptive response to mitigate immune activation is indicated by the upregulation of regulatory markers like CTLA4, but this appears to be inadequate to achieve complete homeostasis [25].

Endometriosis is characterised by a pronounced inflammatory environment that infiltrates the follicular compartment. Alterations in lymphocyte populations include decreased CD4+/CD8+ ratios associated with elevated concentrations of chemokines and cytokines [26]. When granulosa cells are exposed to this cytokine milieu, genes essential for steroidogenesis and follicular response are down-regulated, indicating a connection between immunological dysregulation and cellular functional impairment [10].

Metabolic disorders, including obesity, provoke persistent low-grade inflammation that modifies immune cell infiltration and cytokine production inside the follicle [27]. High body mass index and low embryo quality are associated, which implies that metabolic and immune pathways interact to influence reproductive potential. A notable pattern of reduced follicular production and alterations in immune-cell composition defines poor ovarian response [28]. Macrophage infiltration is diminished, whereas there is enhanced polarisation and proliferation of dendritic cell subsets, indicating dysregulated immunological signalling rather than mere depletion. Immunological dysregulation occurs under these situations, resulting in modified signaling pathways that affect granulosa cell activity, steroidogenesis, and oocyte competence, indicating that immunological equilibrium inside the follicle is essential for reproductive success [23].

The oocyte’s ability to mature into a mature egg depends on a delicate equilibrium between local immunological tolerance and physiological inflammatory signaling, which our findings lend credence to as an immune microenvironment that is constantly maintained inside the ovarian follicle.

## 4. PD-1/PD-L1 Signaling Within the Follicular Compartment

### 4.1. Expression and Distribution of PD-1 and Its Ligands in the Ovary

Mapping PD-1 and its ligands in the ovary uncovers a pattern that deviates from the conventional immune-cell–restricted paradigm seen in peripheral tissues. Expression is located immediately inside the structural components of the follicle (mural and cumulus granulosa cells, as well as the oocyte), suggesting that checkpoint signaling is integrated into the follicular unit rather than being imposed by invading lymphocytes. Local signals, including exposure to cytokines, gonadotropin stimulation, and the hypoxic gradients that occur during follicular expansion, can dynamically regulate PD-L1 expression, as indicated by the transcriptional activity observed in these cells [29].

The granulosa cells seem to be the primary regulators of this system. They are situated at the interface of the vascular compartment and the antral cavity, rendering them susceptible to circulating signals and locally synthesised mediators. The induction of PD-L1 in these cells may be facilitated by interferon-responsive elements via JAK/STAT signalling, namely via the activation of STAT1, which is recognised for upregulating PD-L1 transcription in several cell types [30]. NF-κB pathways can be simultaneously activated under inflammatory conditions, further enhancing increased expression and establishing a direct connection between immune activation and checkpoint upregulation. Hormonal influences presumably impact these processes. Follicle-stimulating hormone and oestradiol modulate transcriptional programs in granulosa cells, which may indirectly govern checkpoint expression via metabolic and signalling intermediates [31].

The localisation inside the cumulus–oocyte complex adds an additional dimension of spatial uniqueness. Cumulus cells form a thick network around the oocyte, facilitating nutrition delivery, providing metabolic support, and transmitting signals. In this compartment, the expression of PD-L1 indicates the establishment of a local immunoregulatory barrier that may restrict the oocyte’s exposure to cytotoxic mediators [32]. The surface expression of PD-L1 on cumulus cells may inhibit the activation of adjacent T cells, hence decreasing the probability of cytokine release near the oocyte. PD-L1 expression derived from oocytes has been suggested as a possible factor in this localised immunoregulatory milieu; however, direct functional evidence inside the human follicular compartment is still scarce [33].

Soluble variants of PD-1 and PD-L1 introduce a diffusible element to this regulatory mechanism. The presence of these molecules in follicular fluid suggests active release mechanisms, such as proteolytic shedding or alternative splicing events that lead to secreted isoforms. The attachment of soluble ligands to PD-1 receptors on T cells may interfere with receptor clustering and subsequent signalling, highlighting the biological importance of concentrations inside the follicular cavity. Consequently, these interactions may facilitate the dissemination of checkpoint-mediated inhibition throughout the follicular fluid and to immune cells that are distal from the initial source, as they are not contingent upon physical contact between cells [34].

Furthermore, the presence of checkpoint proteins in follicular fluid complicates temporal control. Expression is unlikely to remain steady during the follicular period. As the follicle expands, changes in oxygen tension, metabolic requirement, and cytokine production occur, all of which may influence PD-L1 expression [7]. In several tissues, hypoxia-inducible factors, especially HIF-1α, have been shown to control PD-L1 and may also play a role in the follicle, since oxygen gradients are formed during late folliculogenesis. The periovulatory phase is marked by an increase in inflammatory mediators and prostaglandin signalling, which may further influence checkpoint activation, indicating that PD-1/PD-L1 expression may fluctuate in response to physiological transitions rather than being constant [35].

A further consideration is the molecular configuration of checkpoint proteins. Full-length membrane proteins coexist alongside shortened or soluble versions that possess distinct functional consequences. Membrane-bound PD-L1 facilitates direct inhibitory signalling via cell–cell interaction, while soluble variants may function as decoys or modify receptor accessibility [36]. Depending on their relative proportions, these forms may influence the overall intensity and distribution of checkpoint signaling in the follicle. Another method of delivering inhibitory signals in a targeted manner is the enrichment of full-length proteins in extracellular vesicles, which further complicates the landscape [37].

A spatially organized, temporally regulated checkpoint signaling system that is responsive to immune and endocrine inputs is suggested by the integration of these components. Regulation seems to take place at several levels, including transcriptional regulation, post-translational modification, and extracellular dispersion. The complexity of the PD-1/PD-L1 signaling in the ovary suggests that it is unlikely to be a passive reflection of immune infiltration but rather an active regulatory network that is embedded in follicular physiology.

### 4.2. Extracellular Vesicle-Mediated Checkpoint Signaling

PD-L1 in extracellular vesicle populations within follicular fluid represents a signalling mechanism distinctly apart from both membrane-bound contacts and freely soluble proteins. Small extracellular vesicles are generated from endosomal pathways by the invagination of multivesicular structures and then discharged into the extracellular space [8]. During follicular development, granulosa and cumulus cells actively produce vesicles, which are enriched with a specific molecular payload that mirrors the intracellular condition of the donor cell. The presence of PD-L1 in these vesicles may indicate selective loading rather than passive inclusion; however, the exact mechanisms governing this process in the follicular environment remain inadequately understood [38].

Post-translational changes regulating protein localization are likely implicated in the sorting of PD-L1 into vesicles. Ubiquitination, glycosylation, and interactions with adaptor proteins of the endosomal sorting complex needed for transport (ESCRT) machinery may dictate whether PD-L1 is maintained at the plasma membrane or trafficked into vesicular compartments [39]. The interaction with certain tetraspanins, especially vesicles enriched in CD9, indicates that the checkpoint proteins are not randomly dispersed throughout vesicle populations but are selectively localized to specific subtypes. Functional specialisation is suggested by this selectivity, as distinct classes of vesicles may deliver distinct combinations of signalling molecules or target distinct recipient cells [40].

Vesicle-mediated transport facilitates the concentration of PD-L1 in a manner that preserves its structural integrity and receptor binding capability. The lipid bilayer encapsulation protects the protein from enzymatic breakdown in the extracellular environment and extends its functional lifespan relative to soluble versions [41]. Recipient cells may internalise them by direct membrane fusion, receptor-mediated endocytosis, or ligand–receptor interaction at the cell surface. Vesicle-associated PD-L1 may interact with PD-1 on T cells to induce inhibitory signalling without necessitating sustained cell–cell contact, thereby enabling the regulation of immune responses even when far from granulosa or cumulus cells [34].

Vesicle-mediated checkpoint signalling has implications that extend beyond mere suppression of T-cell activation. The administration of PD-L1, in conjunction with co-packaged entities such as microRNAs and signalling proteins, presents the potential for the synchronised control of several pathways in recipient cells [42]. Immune cell uptake may alter transcriptional programs, reduce cytokine production, and influence differentiation towards regulatory phenotypes. The transfer to granulosa cells may influence intracellular signalling networks associated with survival, metabolism, or stress responses, especially if vesicles transport supplementary modulators of the PI3K/AKT or NF-κB pathways. Vesicles in the follicular cavity are spatially distributed, which enables the formation of localised signalling gradients [43].

Vesicles released by mural granulosa cells may disseminate into the antral fluid, whereas those produced by cumulus cells tend to stay localised around the oocyte; nevertheless, direct evidence of spatial vesicle concentration gradients in human follicular fluid is still scarce. This hypothesised segregation may enhance region-specific control of local transmission activity, while the majority of existing data is based on in vitro findings and extrapolations from other biological systems [44]. Additionally, the heterogeneity of signalling is further accentuated by the variability in vesicle size, composition, and surface markers, which is used to adjust checkpoint activity in accordance with the local environmental conditions. The control of vesicle release seems to be influenced by inflammatory and metabolic signals. Exposure to cytokines may enhance vesicle secretion rates and alter cargo composition, while hypoxia and oxidative stress can influence endosomal trafficking routes. Changes in intracellular calcium concentrations and cytoskeletal dynamics also affect vesicle production and release. In pathological situations, such as chronic inflammation or metabolic imbalance, these regulatory processes may lead to a change in vesicle production that either enhances or inadequately compensates for immune activation [45].

Vesicle-mediated signaling is integrated with soluble and membrane-bound checkpoint mechanisms to form a complex regulatory network. Vesicle-mediated signaling may function in conjunction with soluble and membrane-bound checkpoint pathways, thereby enhancing a more extensive immunoregulatory network inside the follicular milieu. Research on PD-L1 signaling in ovarian vesicles is still in its infancy, and what little there is comes mostly from extracellular vesicle profiling, in vitro observations, and generalizations from immune-regulatory mechanisms in other biological systems. Soluble PD-L1 may diffuse over extensive distances, membrane-bound PD-L1 operates locally by direct contact, and vesicle-associated PD-L1 provides targeted delivery with prolonged stability. It is probable that the relative activities of these forms will fluctuate at various phases of follicular development and under varying physiological or pathological conditions. The interaction among these forms dictates the overall function of the checkpoint inside the follicle.

While extracellular vesicle-associated PD-L1 presents a compelling mechanism for localized immune regulation within the follicular context, the current data is mostly indirect, relying on vesicle characterization studies, in vitro findings, and extrapolations from other immune systems. The biological significance of this putative checkpoint network and its direct in vivo functional validation within human ovarian physiology has to be investigated. The lack of uniformity across IVF labs and patient cohorts, as well as variations in vesicle isolation protocols, purification methods, and molecular characterization approaches, makes it technically difficult to interpret signaling associated with extracellular vesicles in reproductive studies.

### 4.3. Functional Impact on T-Cell Activity and Local Immune Tone

The downstream immunological consequences of PD-1 engagement have been well characterised in other immune-regulatory contexts. A similar mechanism may be involved in the modulation of local T-cell activity and inflammatory signalling in the follicular setting [46]. Consequences include diminished IL-2 production, decreased clonal growth, and a transition to anergy or functional fatigue due to prolonged checkpoint engagement [47].

The follicular milieu is influenced by inflammatory cytokines, potentially leading to granulosa cell malfunction and modified steroidogenic activity [47]. The attenuation of interferon-γ via PD-1 signalling diminishes a vital element of inflammatory enhancement, lessening cytokine stress on follicular cells and facilitating controlled immune responses [19,48]. The fluctuation in cytokine release by T cells affects adjacent macrophages and dendritic cells, potentially altering their polarisation state and antigen presentation capabilities. This regulatory network is further tuned by intercellular feedback loops, including IL-10, TGF-β, and other modulators that adjust immune tone without eliminating responsiveness [49].

PD-1 involvement also influences cellular metabolism, a domain that is becoming recognised as fundamental to immune function. Activated T cells must undergo glycolytic reprogramming to facilitate fast proliferation and cytokine synthesis. PD-1 signalling counteracts this metabolic transition by inhibiting the PI3K/AKT/mTOR pathways, resulting in reduced glucose absorption and glycolytic flux, while favouring oxidative phosphorylation [34]. These metabolic limitations inhibit effector activities and encourage a dormant or regulated condition. The modulation of T-cell metabolism may play a role in maintaining a balanced environment conducive to oocyte development within the follicular microenvironment, characterised by fluctuating food availability and metabolic competition across cell types [50].

Checkpoint activity likely indicates changes in follicular physiology over time. Elevated immune activation linked to tissue remodelling and angiogenesis may be permissible during the first phases of follicular development, but the late preovulatory stages may need stricter regulation to safeguard the oocyte throughout its final maturation [51]. Transient elevations in inflammatory mediators occur during ovulation, necessitating measures to avert their escalation into harmful reactions. PD-1/PD-L1 signalling is a versatile mechanism that may adjust the degree of inhibition based on local stimuli, facilitating the precise regulation of immunological activity across several phases of the follicular cycle [52].

The local immune tone results from the integration of checkpoint signalling, cytokine gradients, cellular composition, and metabolic circumstances. The total impact on T-cell behaviour is influenced by variations in ligand availability, receptor expression, and the effectiveness of downstream signalling. Inadequate checkpoint activation may permit prolonged activation and excessive cytokine release, while excessive inhibition might potentially obstruct processes essential for physiological remodelling. Functional balance is maintained via relative control rather than by the absolute degree of activation or repression. Precision is essential in an environment where both immune activation and inhibition are necessary.

### 4.4. Interaction with Intracellular Survival Pathways in Granulosa Cells

PD-L1 expression in granulosa cells has been associated with several survival-related pathways, although direct intracellular signaling functions remain unclear. The activation of the PI3K/AKT axis is recognized as a critical point [9]. Upstream signals, such as autocrine PD-L1 signaling, cytokine activation, or interaction with growth factor receptors, result in the phosphorylation of AKT, hence stabilizing anti-apoptotic proteins and inhibiting pro-apoptotic effectors. Downstream targets such as BAD, FOXO transcription factors, and GSK3β are regulated to promote cell survival, while mTOR activity is sustained to facilitate protein synthesis and cellular growth. The operational integrity of this system is essential during late folliculogenesis, since granulosa cells must sustain a high metabolic output to facilitate oocyte maturation [53].

Inflammatory stress disrupts this equilibrium via the simultaneous activation of JAK/STAT and NF-κB pathways. Interferon-γ signaling triggers the phosphorylation of STAT1 and the transcription of genes associated with immunological activation and death, including proteins that enhance cellular sensitivity to mitochondrial malfunction [54]. Tumour necrosis factor-α (TNF-α) enhances stress responses via NF-κB, leading to increased production of inflammatory mediators and pro-apoptotic proteins, including BAX. Mitochondrial outer membrane permeabilization and cytochrome c release are induced by the convergence of these signals, which initiate the caspase cascade. Some studies have suggested indirect contribution of PD-L1-associated regulation to preservation of AKT activity, mitochondrial integrity and resistance to apoptotic signaling; however, direct intracellular signaling mechanisms in granulosa cells remain incompletely understood [55].

Mitochondrial homeostasis is a critical intersection of immunological signaling and granulosa cell functionality. Reactive oxygen species produced during inflammatory activation may damage mitochondrial DNA and disrupt oxidative phosphorylation, resulting in a depletion of ATP essential for steroidogenesis and cellular maintenance [56]. PD-L1-associated signaling may affect antioxidant responses and maintain electron transport chain efficiency to reduce oxidative stress. Preserving mitochondrial integrity facilitates the survival of granulosa cells and enhances metabolic coupling with the oocyte, which depends on adjacent cells for substrates and regulatory signals throughout maturation [57].

The interaction between checkpoint signaling and intracellular pathways also influences steroidogenic capability. Intact signaling of pathways involving PI3K/AKT and cAMP-dependent mechanisms is essential for the expression and activity of enzymes such as aromatase (CYP19A1) and 3β-hydroxysteroid dehydrogenase. Inflammatory cytokines suppress these enzymes through transcriptional repression and post-translational modifications, resulting in reduced oestradiol synthesis [58]. PD-L1 regulation may indirectly sustain steroidogenesis by maintaining signaling environments that promote the expression and activity of enzymes, hence keeping hormonal circumstances favourable to oocyte competence [9].

Integration with additional signaling network layers in enhanced regulation. Interactions between feedback mechanisms and AKT may influence NF-κB activity and impact the transcription of inflammatory and survival genes. Its interaction with MAPK pathways influences the cellular response to growth factors and stressors [59]. Its interaction with STAT proteins influences the gene expression patterns related to immunological signaling. Phosphorylation, acetylation, and ubiquitination are post-translational changes that regulate the stability and functionality of essential signaling components, facilitating quick adaptability to fluctuations in the follicular environment [60].

**Figure 1:**
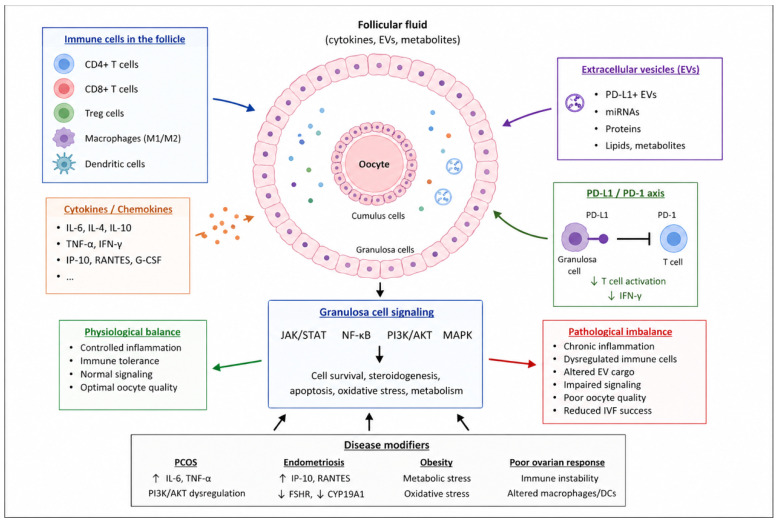
Schematic representation of the follicular milieu and the interactions among immune cells, cytokines, extracellular vesicles, and intracellular signaling pathways.

Granulosa cell metabolic regulation is greatly influenced by these signaling pathways. The PI3K/AKT/mTOR signaling system coordinates glucose absorption, lipid metabolism, and amino acid utilization to fulfil biosynthetic requirements and energy generation. The inflammatory disruption of these pathways leads to a metabolic shift towards catabolic states, diminishing substrate availability for oocyte maintenance [61]. PD-L1-associated signaling may facilitate metabolic balance by reinforcing pathways that enhance anabolic activities and optimize energy use. The dynamic control of intracellular networks necessitates flexibility throughout follicular growth [62]. Granulosa cells must continuously adjust their signaling outputs in response to fluctuations in cytokine levels, hormonal signals, and metabolic inputs. PD-L1 modulation serves as a mechanism for the integration of external immune cues with internal survival pathways, enabling granulosa cells to preserve functionality in circumstances that would otherwise induce cellular stress and decline [63]. A schematic representation of the integrated immune-checkpoint–granulosa cell signaling network within the follicle is shown in Figure 1.

Immune cells in the follicle release cytokines and vesicles carrying proteins, microRNAs, and metabolic regulators. Such signals converge on granulosa cells, activating pathways including JAK/STAT, NF-κB, PI3K/AKT, and MAPK. This network maintains immunological homeostasis and enhances oocyte competency under physiological settings. Impaired granulosa cell function due to pathological conditions of chronic inflammation, modified vesicle cargo, and dysregulated signaling results in diminished oocyte quality and decreased reproductive success.

## 5. Granulosa Cells as Immune-Responsive Targets

### 5.1. Granulosa Cells Beyond Steroidogenesis

Granulosa cells are essential mediators of the follicular milieu, converting endocrine, metabolic, and immunological signals into functional outcomes that directly influence oocyte development. Traditionally, they have been characterized by their involvement in steroid hormone synthesis, especially the production of oestradiol through aromatase activity [64]. However, mounting evidence indicates a wider functional range encompassing cell cycle regulation, cytoskeletal organization, metabolic support, and intercellular communication within the follicle. Transcriptional profiling reveals a coordinated expression of genes associated with mitotic spindle construction, chromosomal segregation, and checkpoint regulation, reflecting a dynamic state that continuously adapts to the developmental stage of the follicle [1].

Immune-derived mediators provide an additional regulatory layer that alters granulosa cell activity at several levels. The cytokine receptors on these cells enable direct responses to cytokine signals, including interferon-γ, tumour necrosis factor-α, and interleukins, which activate intracellular signaling pathways independent of endocrine signaling mechanisms [63]. The activation of JAK/STAT pathways induces alterations in transcriptional programs related to immune response and apoptosis, whereas the involvement of NF-κB pathways amplifies the production of inflammatory mediators and stress-response genes. These signals integrate and modify the functional phenotype of granulosa cells, influencing proliferation, differentiation, and survival [65].

The interaction between immunological signaling and gonadotropin response represents a significant vulnerability. The expression of follicle-stimulating hormone receptors is meticulously controlled and is crucial for sustaining steroidogenic function and follicular development [66]. Exposure to cytokines has been shown to downregulate FSHR expression, diminishing susceptibility to gonadotropin stimulation and altering downstream signaling via cAMP-dependent pathways. Disruption of this axis results in decreased production of oestradiol and adversely impacts the hormonal environment necessary for oocyte development. The concurrent manipulation of enzymes like CYP19A1 further disrupts steroidogenesis, establishing a clear connection between immune activation and endocrine dysfunction [56].

Signals coming from the immune system also affect the structural integrity of granulosa cells. The organization of the cytoskeleton, particularly the microtubule networks that facilitate mitotic spindle formation, is crucial for effective cell division and the preservation of follicular architecture. We have noted modifications in gene expression related to spindle assembly and cell cycle checkpoints after prolonged in vitro culture and inflammatory stress, indicating that immunological signaling may fundamentally affect cellular architecture. Disruption of these mechanisms may result in atypical proliferation, alterations in differentiation status, or the development of traits linked to cellular stress responses [2].

Granulosa cells biologically sustain the oocyte via well-regulated mechanisms that govern glucose absorption, glycolysis, and amino acid metabolism. Immune activation disrupts these processes by modifying signaling pathways such as PI3K/AKT and AMPK, thereby influencing energy balance and substrate availability. Oxidative stress and inflammatory signaling disrupt mitochondrial function, hence diminishing metabolic efficiency. Decreased ATP synthesis and the buildup of reactive oxygen species may impair granulosa cells’ capacity to fulfil the metabolic requirements of the oocyte, particularly in the last stages of maturation [67].

### 5.2. PD-L1 Dependent Regulation of Granulosa Cell Apoptosis and Survival Pathways

Intracellular survival control is associated with inflammatory sensing through the expression of PD-L1 in granulosa cells. Induction occurs via IFN-induced STAT1 activation, often with TNF-α-mediated signaling, creating a transcriptional milieu favourable for checkpoint expression under stress conditions [68]. Localization is not confined to the plasma membrane; intracellular reservoirs in endosomal and cytoplasmic compartments indicate a function in signaling processes beyond immune cell interactions. Trafficking pathways and post-translational modifications determine the distribution of PD-L1 between intracellular and surface compartments, suggesting that its spatial location within the cell may influence its context-dependent effects. The presence of intracellular vesicles containing PD-L1 suggests possible interactions with signaling complexes that modulate kinase activity and protein stability, although direct intracellular signaling functions in granulosa cells have not been well established [69].

Current research suggests an association between PD-L1 expression and PI3K/AKT-related survival signaling in granulosa cells, although direct biochemical interactions have not been demonstrated. By inhibiting mediators such as BAD and favouring the stabilization of BCL-2 family members that maintain mitochondrial integrity, the activation of AKT results in a shift in the balance of pro- and anti-apoptotic proteins [70]. Inhibition of the FOXO transcription factor diminishes the transcription of genes associated with oxidative stress and apoptotic signaling, hence decreasing cellular susceptibility under inflammatory circumstances. Moreover, mTOR activation augments protein synthesis, ribosomal activity, and metabolic activities essential for sustaining granulosa cell function [71]. Changes in the phosphorylation status of AKT concurrently affect many downstream pathways, regulating cell cycle progression, metabolic control, and stress signal resistance. In experimental models of PCOS, the elevation in PD-L1 reinstated AKT activity and reduced apoptotic markers, suggesting that checkpoint-associated signaling directly influences survival pathways rather than being exclusively mediated via immune-cell suppression [9].

Chronic inflammatory signaling may disrupt granulosa cell homeostasis via many previously described mechanisms, ultimately leading to cellular stress and apoptosis [54]. In a feed-forward loop created by the interaction of various signaling networks, inflammatory mediators intensify cellular damage. PD-L1-associated signaling influences this development by maintaining the functionality of survival pathways and preventing the full activation of apoptotic mechanisms, hence affecting the threshold at which granulosa cells experience programmed cell death [9].

Reactive oxygen species contribute to the exacerbation of cellular stress during inflammatory activation. Mitochondrial dysfunction and the activation of enzymatic sources, such as NADPH oxidases, lead to elevated ROS production and oxidative modifications of lipids, proteins, and nucleic acids [72]. Oxidative stress accumulation affects granulosa cells’ energy-dependent functions by reducing ATP production and impairing mitochondrial respiration. Alterations in redox state also influence signaling pathways, including apoptosis and inflammation [73]. PD-L1-associated effects on mitochondrial stability and redox balance may reflect broader interactions between inflammatory signaling and cellular stress responses.

The regulation of antioxidant systems, particularly glutathione-dependent pathways, may aid in preserving cellular function when inflammatory signaling may otherwise result in metabolic failure [74].

Integrity of intracellular signaling pathways that are susceptible to immune-derived stress is essential for the steroidogenic activity. Coordinated regulation via PI3K/AKT and cAMP-mediated pathways is essential for the expression and enzymatic activity of aromatase and other steroidogenic enzymes. Inflammatory cytokines modify transcriptional regulation and enzymatic function, resulting in reduced oestradiol synthesis and disrupted follicular hormone equilibrium [20]. The upkeep of signaling pathways linked to PD-L1 activity sustains the production of steroidogenic enzymes, hence conserving the hormonal environment essential for oocyte maturation. The functional capacity of granulosa cells is maintained by the crosstalk between survival signaling and endocrine pathways, ensuring that the follicular development is able to continue in the presence of inflammatory mediators.

The assessment of follicular cytokine profiles is challenging because of significant variability in cytokine concentrations influenced by stimulation procedures, metabolic conditions, infertility diagnosis, follicular size, timing of sample collection, and general patient heterogeneity.

### 5.3. Cytokine-Driven Modulation of Granulosa Cell Function and Oocyte Competence

Exposure of granulosa cells to cytokines in follicular fluid alters transcriptional pathways that govern differentiation, steroidogenesis, and response to gonadotropins. Chemokines like CXCL10 and CCL5, together with cytokines such as interferon-γ, TNF-α, IL-6, and G-CSF, provide signaling inputs that converge on receptors located on the surface of granulosa cells [19]. Ligand binding triggers intracellular cascades, mostly controlled by the JAK/STAT and NF-κB pathways, which swiftly alter gene expression patterns. Reduced synthesis of oestradiol, reduced sensitivity to follicle-stimulating hormone, and reduced transcription of the FSH receptor and aromatase are examples of downstream consequences. Alterations in promoter accessibility and transcription factor recruitment appear to be the mechanisms by which these effects are mediated, thereby directly linking inflammatory signaling to endocrine disruption [59].

The interplay of inflammatory signaling pathways in granulosa cells is probably tightly interconnected and context-dependent, and some of these mechanisms have already been described in previous sections [75]. MAPK signaling participates in cell proliferation, differentiation, and stress responses via the ERK and p38 pathways. Crosstalk between these pathways regulates the functional activity of granulosa cells, which either maintain their activity or transition to a stress-responsive phenotype with altered cell-cycle dynamics and reduced steroidogenesis.

Metabolic reprogramming is accompanied by these transcriptional and epigenetic modifications. Cytokine signaling influences glucose absorption, glycolytic flux, and mitochondrial respiration in granulosa cells, redirecting energy production towards pathways that facilitate inflammatory responses instead of anabolic activities [76]. The diminished efficacy of oxidative phosphorylation restricts ATP availability, whereas the accumulation of metabolic intermediates may influence signaling pathways via feedback mechanisms. Lipid metabolism is modified, with alterations in fatty acid oxidation and lipid storage impacting membrane composition and signaling efficacy. Granulosa cells’ capacity to supply substrate for oocyte maturation is altered by these metabolic modifications [61].

Paracrine signaling between granulosa cells and the oocyte is particularly susceptible to interference by cytokines. Connexins create gap junctions that facilitate the flow of ions, metabolites, and regulatory chemicals, hence preserving synchronization between somatic cells and the oocyte. Cytokines may modify connexin expression and channel functionality, resulting in diminished intercellular connectivity. Disruption of coordinated signaling impairs meiotic progression and cytoplasmic maturation of the oocyte, resulting in abnormalities that may not be morphologically apparent but influence developmental competence [77].

Extracellular vesicles produced under inflammatory circumstances possess a changed molecular payload that reflects the cytokine environment. The microRNA concentration, protein composition, and lipid profiles are altered in the receiving cells inside the follicle [78]. By internalizing these vesicles, granulosa cells or the oocyte can modify gene expression and signaling pathways, thereby amplifying the effects of soluble cytokines. Consequently, vesicle-mediated transfer functions in conjunction with direct cytokine signaling to expand and prolong the inflammatory effects inside the follicular milieu [42]. The interaction of these processes results in a coordinated regulation of granulosa cell activity that surpasses the aggregate of individual signaling events. The cumulative impacts on transcription, metabolism, intercellular communication, and signaling alter the circumstances of oocyte development and affect the oocyte’s ability to mature and facilitate future embryonic development.

### 5.4. Integration of Immune Signaling with Oocyte Competence and Developmental Potential

Alterations in granulosa cell activity, follicular metabolism, and the biochemical composition of follicular fluid convert immune-derived signals into quantifiable impacts on oocyte competence. Cytokine exposure alters the transcriptome of granulosa cells, affecting the nuclear and cytoplasmic development of the oocyte [4]. Reduced expression of the FSH receptor and aromatase modifies oestradiol synthesis and disturbs the hormonal gradients that govern meiotic development. Simultaneously, signaling pathways induced by inflammatory mediators affect the production of growth factors and metabolites that are sent to the oocyte via gap junctions and paracrine interactions. These alterations affect the accumulation of transcripts and proteins essential for early embryonic development, a process mostly reliant on maternal reserves deposited during oogenesis [79].

Cytoplasmic maturation is particularly susceptible to variations in the metabolic support offered by adjacent cells. Granulosa cells provide substrates such as pyruvate, amino acids, and nucleotides that sustain oocyte metabolism. The cytokine-induced impairment of glycolysis and mitochondrial activity diminishes the availability of substrates, thereby altering ATP generation and redox balance inside the oocyte. Mitochondrial activity indirectly influences the oocyte by altering the surrounding microenvironment, which alters the distribution and function of mitochondria throughout maturation. Although fertilisation may not be impeded by these metabolic derangements, they may impede subsequent embryonic development by disrupting cellular organization and energy production [80].

Immune signaling influences the control of meiotic spindle assembly and chromosomal segregation. Granulosa cells modulate ionic equilibrium, provide metabolic support, and secrete signaling chemicals to alter the milieu necessary for optimal spindle construction [81]. Disruption of these inputs may result in anomalies in spindle structure, hence elevating the probability of chromosomal misalignment or aneuploidy. Alterations in the cytoskeletal dynamics of granulosa cells, prompted by inflammatory signaling pathways, may also exacerbate instability in the oocyte microenvironment. These findings highlight a correlation between immune-mediated alterations and essential mechanisms that preserve genomic integrity [82].

Supplementary communication routes via extracellular vesicles to transmit immune-related signals to the oocyte. Vesicles released by granulosa and cumulus cells comprise microRNAs, proteins, and lipids that influence gene expression and signaling pathways in target cells. In inflammatory situations, the composition of vesicle cargo is altered to favour molecules linked to stress responses and immune modulation. The internalisation of these vesicles by the oocyte may potentially impact transcript stability, protein synthesis, and intracellular signaling, thereby affecting developmental competence beyond acute cytokine exposure.

## 6. Disease-Specific Modulation of the Follicular Immune-Checkpoint Axis

### 6.1. Polycystic Ovary Syndrome

PCOS establishes an inflammatory and metabolic signalling environment that converges on granulosa cells, resulting in transcriptional, receptor and intracellular trafficking alterations [83]. Certain inflammatory and immunological regulatory systems have been addressed in previous sections. However, granulosa cell function and oocyte developmental competence may be further compromised by their prolonged activation in a metabolically changed follicular milieu in PCOS [84]. Then, there is the intracellular havoc of insulin resistance. Dysregulated downstream indicating outputs may result from aberrant stimulation of PI3K pathways by hyperinsulinemia, which would normally crosstalk with gonadotropin-mediated intracellular regulation [85]. In PCOS granulosa cells, altered insulin signaling may affect PI3K/AKT pathway activity and contribute to impaired metabolic coordination. Increased lipid storage in the cell changes the membrane microdomains, which alters the location of receptors and the strength of signalling. The crosstalk between insulin receptor signalling and the inflammatory pathways maintains the activation of NF-κB and STAT networks, and consolidates transcriptional programs that promote cytokine production and cellular stress responses. Altered AMPK activity further impairs energy sensing, limiting the ability of granulosa cells to adapt to fluctuating metabolic demands during follicular growth [86].

In this context, PD-L1 expression is the result of integration of these signalling pathways at the transcriptional and post-translational level. STAT1 directly binds the PD-L1 promoter and upregulates gene expression following interferon signalling, while NF-κB contributes to additional transcriptional input in inflammatory conditions [30]. PD-L1 glycosylation stabilises the protein and increases its persistence at the cell surface, while modulation of GSK3β activity affects ubiquitination and degradation rates. Functional PD-L1 output is modulated by redistribution of PD-L1 between membrane and intracellular compartments to engage PD-1 on immune cells and also as a component of intracellular signalling complexes. Interaction with PI3K/AKT pathways enhances survival signalling and reduces susceptibility to apoptosis under chronic inflammatory stress [87].

The immune cell composition of PCOS follicles reflects altered recruitment and differentiation of immune cells by local cytokine gradients. Monocytes in these conditions adopt phenotypes with increased expression of inflammatory mediators and changes in metabolic profiles which lead to sustained cytokine production [3]. T-cell populations display changes in activation status with increased expression of inhibitory receptors such as PD-1 and CTLA4, suggesting chronic antigenic stimulation. Differentiation toward regulatory phenotypes might occur in response to persistent signalling, but the functional capacity of these cells is still dictated by the surrounding inflammatory milieu. Feedback loops are generated by cytokine-driven interactions between immune cells and granulosa cells to maintain local signalling intensity [88].

EV signalling plays a role in the dissemination of these molecular alterations. Granulosa cell secreted vesicles have a different cargo composition in an inflammatory environment including microRNAs targeting genes related to steroidogenesis, apoptosis and metabolic pathways [89]. The uptake of these vesicles by neighbouring cells reinforces transcriptional and signalling changes, extending the influence of the inflammatory environment beyond direct cytokine exposure. The transfer of regulatory molecules by vesicles is an additional modulation of intracellular pathways such as those involved in the control of AKT phosphorylation and NF-κB activity, further integrating immune and metabolic signalling within the follicular compartment.

Disruption of intracellular coordination between these pathways changes granulosa cell function on several levels. The biochemical environment experienced by the oocyte and consequently the accumulation of maternal factors necessary to subsequent developmental processes includes receptor signalling, transcriptional control, metabolic regulation and intercellular communication.

### 6.2. Endometriosis

Endometriosis creates a follicular milieu characterised by persistent inflammatory signalling, altered chemokine gradients, and oxidative stress, which adversely affect granulosa cell activity and oocyte maturation [90]. Increased concentrations of chemokines (e.g., CXCL10 [IP-10] and CCL5 [RANTES]) and cytokines (e.g., G-CSF and IL-6) indicate the activation of interferon-responsive and NF-κB-dependent pathways. CXCL10 interacts with CXCR3 receptors on granulosa cells, initiating downstream signalling cascades including JAK/STAT and MAPK pathways, resulting in the transcriptional suppression of FSH receptor and CYP19A1. A changed hormonal environment for synchronised follicular development results from reduced expression of these genes, which restricts response to gonadotropins and hinders oestradiol production [91].

The molecular landscape is mostly influenced by interferon-γ signalling. Activation of STAT1 results in the transcription of genes that are involved in immune activation and apoptosis, as well as an increase in the expression of chemokines that maintain the recruitment of immune cells [92]. TNF-α activates NF-κB, stabilises transcripts of inflammatory mediators, and stimulates the synthesis of additional cytokines in the follicular compartment. Inflammatory signalling is amplified and a transcriptional program that prioritises cellular stress over differentiation is reinforced by the concerted activity of these pathways. Further regulation of transcription factor activity and gene expression associated with apoptosis and cytoskeletal organization is achieved through the activation of the p38 MAPK and JNK pathways [93].

Oxidative stress is a significant characteristic of the endometriotic follicular milieu. Mitochondrial malfunction and inflammatory stimulation of enzymatic sources result in increased generation of reactive oxygen species and damage to cellular components [94]. Oxidative stress buildup disrupts oxidative phosphorylation and mitochondrial DNA integrity, reducing ATP availability essential for steroidogenesis and cellular maintenance. Alterations in redox equilibrium also influence signalling pathways, including those regulating apoptosis and inflammatory responses. Granulosa cells subjected to these circumstances exhibit reduced viability and modified metabolic activity, thereby impairing their capacity to facilitate oocyte formation [95].

The dynamics of immune cells inside the follicle also influence these processes. Decreased CD4^+^/CD8^+^ ratios indicate a transition towards cytotoxic T-cell predominance, accompanied by heightened synthesis of interferon-γ and other pro-inflammatory agents. Macrophage populations exhibit features linked to chronic inflammatory activation and release cytokines and growth factors that influence both immune and non-immune cells. Immune cells and granulosa cells establish a microenvironment with persistent signalling, rather than transient activation, which maintains high cytokine levels during follicular growth [96].

Extracellular vesicles originating from granulosa and immune cells possess molecular markers indicative of an inflammatory condition. Altered microRNA profiles in these vesicles influence genes involved in steroidogenesis, apoptosis, and metabolic control. The absorption of vesicles by granulosa cells modifies intracellular signalling pathways and amplifies transcriptional alterations induced by soluble cytokines. Gene expression and protein synthesis may be impacted by the vesicle cargo transfer to the oocyte, which may have an impact on cytoplasmic maturation and developmental competence [97].

Modifications in these interconnected pathways affect the morphology and functionality of the follicle. The disruption of cytoskeletal arrangement in granulosa cells hinders their capacity to preserve follicular architecture and sustain the oocyte. Impaired gap junction communication disrupts the transport of metabolites and signalling chemicals, hence affecting synchronisation between somatic cells and the oocyte. Conditions that disrupt the typical progression of follicular development at multiple levels are the result of the combined actions of cytokine signalling, oxidative stress, and altered intercellular communication.

### 6.3. Obesity and Metabolic Inflammation

Obesity creates a follicular environment that simulates metabolic surplus and immunological signalling. Granulosa cells are subjected to elevated insulin levels, increased free fatty acids, and modified adipokine concentrations [50]. Activation of the insulin receptor and IGF-1 receptor engages PI3K signalling; however, downstream coordination is compromised. AKT phosphorylation persists without adequate integration with metabolic balance. Lipid buildup inside the cell alters the membrane structure, namely lipid rafts, hence affecting receptor clustering and signal transmission [98].

Free fatty acids are translocated into mitochondria via CPT1-mediated transport, hence enhancing β-oxidation. Augmented electron transport chain activity, heightened electron leakage, and elevated generation of reactive oxygen species. Antioxidant systems, such as glutathione and superoxide dismutase, are surpassed [99]. Subsequently, mitochondrial DNA sustains damage, resulting in a decline in the efficacy of oxidative phosphorylation. ATP generation becomes inconsistent, impacting functions that need a steady energy supply, such as steroid synthesis and intercellular signalling [100].

Subsequently, introduce cytokine exposure. IL-6 and TNF-α initiate the JAK/STAT and NF-κB signalling pathways. Phosphorylation of STAT3 alters the transcription of genes associated with metabolism and inflammation. NF-κB transcriptionally enhances the expression of inflammatory mediators and stabilises their mRNA. Simultaneously, the suppression of FSH receptor signalling reduces cAMP synthesis. Reduced PKA activity restricts the transcription of CYP19A1, hence impairing aromatase function and oestradiol production [101].

Leptin signalling also alters this network. Its receptor interacts with JAK2/STAT3, enhancing inflammatory transcriptional pathways. Elevated leptin also influences PI3K pathways, impacting glucose absorption and cellular metabolism. Adiponectin signalling, which activates AMPK and facilitates metabolic equilibrium, is diminished [102]. The diminished activation of AMPK hinders fatty acid oxidation efficiency and disrupts energy sensing, resulting in chronic metabolic stress. Immune-cell infiltration is indicative of these circumstances. The increase in CD4+ T cells and cytokine production maintains the activation of STAT and NF-kB. Macrophages transition to cytokine-secreting phenotypes rather than engaging in tissue healing. A consistent signal is emitted by these cells, which maintains an inflammatory state in the follicle. The interaction between immune cells and granulosa cells enhances the activation of intracellular pathways [103].

In this context, extracellular vesicles released include microRNAs that target components of insulin signalling, genes involved in lipid metabolism, and regulators of mitochondrial activity. In the recipient granulosa cells, the gene expression is altered by the uptake of these vesicles. This is followed by modifications in AKT signalling, NF-κB activity, and the expression of metabolic enzymes. Comparable vesicles may interact with the oocyte, affecting mitochondrial distribution and redox status during maturation.

A further disadvantage of the majority of contemporary reproductive immunology research is the small sample sizes and limited repeatability across various IVF groups. Substantial diversity in patient characteristics, ovarian reserve, stimulation protocols, metabolic profiles, and laboratory techniques may influence the observed immunological and inflammatory biomarkers. Therefore, prudence is essential when attempting to generalise mechanistic results across reproductive groups.

## 7. Extracellular Vesicles as Carriers of Immune and Checkpoint Signals Within the Follicle

### 7.1. Biogenesis and Molecular Sorting of Follicular Extracellular Vesicles

Early endosomes develop into late endosomes, and then become multivesicular bodies, when the endosomal system starts the process of extracellular vesicle production. Intraluminal vesicles originate from the inward budding of the endosomal membrane, a process necessitating cargo selection and synchronised membrane deformation. ESCRT-independent pathways operate concurrently under certain cellular circumstances [104]. Neutral sphingomyelinase produces ceramide, which increases membrane curvature by altering lipid packing, facilitating vesicle budding without the involvement of classical ESCRT components. Tetraspanins, including CD9, CD63, and CD81, arrange distinct membrane microdomains that function as scaffolds for cargo selection [105]. Tetraspanin-rich domains aggregate receptors, signalling molecules, and adhesion molecules, effectively pre-sorting cargo prior to vesicle formation. This structure is enabled by cholesterol-enriched lipid rafts that stabilise membrane regions with unique physicochemical characteristics, allowing the selective integration of proteins based on their affinity for these environments [106].

The selection of cargo is rigorously regulated and mirrors the intracellular signalling condition of the cell. Ubiquitination functions as a primary sorting signal that channels proteins into intraluminal vesicles. Deubiquitinases may reverse this process, salvaging certain proteins from degradation and facilitating their return to the plasma membrane [107]. Glycosylation influences protein folding and stability, as well as retention within membrane domains in anticipation of vesicle incorporation. Phosphorylation events provide docking sites for adaptor proteins like ALIX and TSG101, which connect the cargo to the ESCRT machinery. Proteins containing PDZ domains enhance this process by tethering specific cargos to sorting platforms, facilitating selective rather than indiscriminate membrane engulfment [108].

RNA loading provides an additional dimension of selectivity. Small RNAs, like microRNAs, are preferentially encapsulated in vesicles via interactions with RNA-binding proteins. hnRNPA2B1 identifies sequence patterns and facilitates the transport of certain microRNAs into vesicles, while YBX1 stabilises RNA complexes and enhances their integration [109]. AGO2 participates in RNA-induced silencing complexes and contributes to sorting in certain contexts. Cellular stress alters the expression and activity of RNA-binding proteins, modifying the RNA cargo profile to include molecules associated with inflammation, metabolism, or apoptosis. Vesicles may influence gene expression in receiving cells persistently by selective RNA packaging [110].

Cytoskeletal components are essential for vesicle formation and transport. Actin remodelling generates membrane tension and facilitates the development of budding sites. Microtubules function as conduits for the transport of multivesicular entities to the plasma membrane. GTPases of a modest size regulate these processes with a high degree of specificity [111]. ARF6 regulates membrane morphology and endosomal transport, whereas Rho family GTPases govern actin structure. Rab proteins determine trafficking pathways and docking characteristics. Rab27a and Rab27b facilitate the positioning of multivesicular bodies near the plasma membrane and regulate vesicle exocytosis. Rab11 and Rab35 govern recycling pathways and the equilibrium of secretion. SNARE complexes facilitate the ultimate membrane fusion, enabling vesicles to be discharged into the extracellular environment [112].

Intracellular calcium concentrations regulate this mechanism at various stages. Temporary elevations in Ca^2+^ facilitate the fusion of multivesicular structures with the plasma membrane by activating calcium-sensitive proteins that mediate membrane docking [113]. Consequently, protein kinase C facilitates this control by phosphorylating elements of the fusion apparatus. Phospholipases alter the lipid content of membranes to promote curvature and fusion. Cellular alterations are often triggered by hormone signalling pathways, particularly those involving G protein-coupled receptors that function via second messengers such as IP3 and DAG, therefore connecting endocrine signalling to vesicle release.

Metabolic and environmental factors significantly impact vesicle biogenesis. Hypoxia stimulates HIF-1α, which governs the transcription of genes associated with endosomal trafficking, such as Rab proteins and elements of the ESCRT machinery [114]. Acidic microenvironments may modify enzyme activity and membrane characteristics, hence affecting sorting efficiency. Oxidative stress induces alterations in lipids and proteins, impacting their integration into vesicles. Mitochondrial failure reduces ATP availability, hence constraining the energy-dependent processes of vesicle production and transport. Together, these factors regulate the outflow of vesicles in accordance with the follicle’s physiological state [115].

Differential vesicular molecular profiles of granulosa and cumulus cells. Variations in transcriptional activity and intracellular signalling lead to distinct cargo compositions, with cumulus cells favouring the packing of molecules associated with oocyte support, whereas granulosa cells generate vesicles rich in signalling intermediates and metabolic regulators. Cytokine exposure further diversifies vesicle populations by altering the expression of adaptor proteins and sorting mechanisms. The resultant variability illustrates the dynamic interaction among immunological signalling, metabolic status, and intracellular trafficking, yielding vesicle populations customised to the unique circumstances inside each follicle [116].

### 7.2. PD-L1 Packaging and Checkpoint Signal Dissemination

PD-L1 is integrated into extracellular vesicles via controlled intracellular trafficking rather than by passive membrane shedding. PD-L1 is synthesised in the endoplasmic reticulum, processed in the Golgi apparatus, and then transported to the plasma membrane by vesicular transport [117]. A portion of the protein is subsequently internalised by clathrin-dependent and clathrin-independent endocytosis. Endocytosed PD-L1 is transported to early endosomes, where sorting determinations occur. A portion of the protein is returned to the membrane, while another portion is sent to multivesicular bodies for sorting into intraluminal vesicles [118].

Post-translational changes are essential to the process. Glycosylation enhances the stability of PD-L1 and inhibits its proteasomal degradation, hence augmenting its accessibility for vesicular sorting. Regulation of trafficking adaptor contacts that dictate PD-L1 membrane retention or internalisation by phosphorylation [119]. Ubiquitination functions as a sorting signal within the endosomal system, directing PD-L1 to ESCRT-dependent pathways. The state of ubiquitination is modulated by the activity of kinases such as GSK3β, hence linking the turnover of checkpoint proteins with intracellular signalling conditions. The proportion of PD-L1 designated for sorting into extracellular vesicles or for breakdown and recycling is determined by the equilibrium of these changes.

The refinement of PD-L1 packing is associated with tetraspanin-enriched microdomains. CD9, CD63, and CD81 delineate membrane domains that function as platforms for vesicle biogenesis. PD-L1 molecules located in these domains are selectively encapsulated into vesicles during the inward budding of the endosomal membrane. PD-L1 may interact with the ESCRT machinery via adaptor proteins, including ALIX and syntenin. The lipid makeup of these microdomains, particularly their abundance in cholesterol and sphingolipids, stabilises protein clustering and facilitates effective sorting into vesicles [120].

PD-L1 is encapsulated and presented on the vesicle surface in a manner that maintains its extracellular domain. This structural conservation facilitates interaction with PD-1 receptors on target cells subsequent to vesicle release. Vesicle-associated PD-L1 does not need cell-to-cell interaction to exert its effects [121]. The engagement of PD-1 on T cells recruits SHP-2 phosphatase, causing the dephosphorylation of signalling intermediates and attenuation of TCR signalling. Inhibiting the PI3K/AKT and MAPK pathways reduces cytokine synthesis and cell proliferation, resulting in T cells transitioning to a less activated state.

Follicular-fluid dynamics may facilitate the wider distribution of checkpoint-associated signals within the follicular environment. Vesicles disseminate throughout the antral cavity and engage with immune cells at varying distances from their source. Vesicle-associated forms exhibit greater stability than soluble PD-L1 due to the protective nature of the lipid bilayer. This facilitates extended signalling and reduces vulnerability to proteolytic degradation. Moreover, vesicle-mediated transport increases the accumulation of PD-L1 at the interaction locus, enhancing the efficacy of receptor binding [122].

Granulosa cells may internalise vesicles expressing PD-L1, transporting checkpoint proteins into intracellular compartments. Uptake is enabled via receptor-mediated endocytosis or membrane fusion, depending upon the vesicle’s composition and surface indicators [42]. Internalised PD-L1 may potentially interact with signalling pathways that regulate survival and metabolism, connecting extracellular checkpoint signals to intracellular signalling. Such connections may influence AKT phosphorylation, mitochondrial integrity, and resistance to apoptosis under inflammatory circumstances [123].

Every stage in this pathway is influenced by cytokine exposure. Interferon-γ stimulates PD-L1 transcription and enhances its accessibility for vesicular inclusion. TNF-α regulates endosomal trafficking and facilitates vesicle release via alterations in cytoskeletal dynamics and membrane fusion processes [124]. Hypoxia also regulates HIF-1α-dependent transcription and endosomal sorting mechanisms that control PD-L1 localisation. Collectively, these parameters influence the quantity and functional status of PD-L1 in extracellular vesicles, connecting checkpoint spreading to the inflammatory and metabolic condition of the follicle [125].

### 7.3. MicroRNA and Protein Cargo Shaping Granulosa Cell Signaling

Extracellular vesicles produced in follicular fluid contain a selective payload of microRNAs that directly influence gene expression in recipient granulosa cells. These microRNAs are not included arbitrarily. RNA-binding proteins like hnRNPA2B1 identify certain sequence patterns and transport particular microRNAs into vesicles [126]. YBX1 stabilises and facilitates the integration of RNA complexes, while AGO2 interacts with microRNA-mRNA complexes and aids in their sorting under stress circumstances. As a consequence of cytokine exposure, the expression and activity of these RNA-binding proteins are altered, leading to a shift in the cargo of vesicles toward microRNAs that are linked to metabolic regulation, apoptosis, and inflammation [127].

Upon delivery to recipient cells, microRNAs exert their effects by sequence-specific binding to target mRNAs, leading to translational suppression or mRNA destruction. MicroRNAs that target components of the PI3K/AKT pathway reduce AKT phosphorylation and subsequent signalling activity, hence affecting cell survival and metabolic function [128]. Others focus on MAPK pathway constituents, altering ERK and p38 activation, hence affecting proliferation and stress responses. MicroRNAs that target messenger RNAs for the FSH receptor or steroidogenic enzymes diminish response to gonadotropins and impair oestradiol synthesis. The effects are enduring, since microRNA-mediated control persists after the initial absorption [129].

The protein payload inside vesicles introduces an additional dimension of regulatory intricacy. Signalling proteins encapsulated in vesicles retain their functional activity and may directly engage with intracellular pathways upon release. Kinases and phosphatases regulate the phosphorylation states of target proteins, influencing the PI3K/AKT, NF-κB, and JAK/STAT signalling pathways [65]. Metabolic enzymes may alter the local substrate availability, hence affecting glycolysis, lipid metabolism, and redox equilibrium. Structural proteins facilitate the organization of the cytoskeleton, which defines cell shape and promotes intercellular communication.

The coordination of microRNA and protein cargo allows the simultaneous control of numerous pathways. MicroRNAs inhibit the synthesis of certain proteins, while proteins transported by vesicles may rapidly alter signaling states. This dual strategy facilitates rapid and enduring modifications in cellular behaviour. Additionally, these components may interact to enhance their effects. The simultaneous suppression of regulatory inhibitors and activation of signaling proteins yields a more significant alteration in the pathway than either process independently [130].

Variations in vesicle cargo composition generated by cytokines reflect the intracellular signaling levels of the donor cells. The activation of NF-κB and STAT pathways enhances the synthesis of microRNAs that govern inflammation, which are then encapsulated in vesicles. Hypoxia and oxidative stress alter RNA and protein cargo via adjustments in transcriptional programs and post-translational alterations. Consequently, modified cargo composition serves as a molecular hallmark of the follicular environment, encapsulating information about immunological activity and metabolic condition [78].

The absorption of vesicle cargo by granulosa cells gradually impacts intracellular signaling networks. Repeated exposure to vesicles with analogous cargo amplifies pathway activation or inhibition, leading to enduring alterations in gene expression and cellular function. Differences in granulosa cell behaviour across the follicle are a result of the integration of these signals, which affect apoptosis, metabolism, and the response to hormonal stimulation.

### 7.4. Target Cell Interaction and Uptake Mechanisms

Extracellular vesicles interact with destination cells via specialized molecular interfaces rather than via random collisions. Surface proteins on vesicles that dictate docking specificity include integrins, tetraspanins, and heparan sulfate-binding domains. The first level of contact involves the binding of the ligand to the receptor at the target cell membrane [131]. Vesicular integrin profiles may influence preferred binding to certain cell types, whereas tetraspanin complexes enhance these interactions by organizing membrane microdomains. Heparan sulphate proteoglycans on granulosa cells serve as a principal attachment point, localizing vesicles on the cell surface and facilitating subsequent absorption [42].

Various uptake mechanisms function based on the vesicle composition and the condition of the target cell. Clathrin-mediated endocytosis is characterized by the recruitment of adaptor proteins, such as AP2, the creation of coated pits, and their subsequent internalization into early endosomes [132]. Caveolin-dependent pathways rely on cholesterol-enriched lipid raft domains and facilitate vesicle entrance via flask-shaped membrane invaginations. Macropinocytosis is a non-specific uptake mechanism characterized by actin-mediated membrane ruffling and the generation of enormous vesicles that internalize external substances. Receptor-mediated identification of vesicular surface molecules may enhance phagocytic ingestion by immune cells, particularly macrophages [133].

Subsequent to internalization, the vesicles are transported to endosomal compartments where sorting determinations occur. Fusion with early endosomes liberates cargo into the cytoplasm, whereas advancement to late endosomes and lysosomes may result in destruction. MicroRNAs and proteins may traverse endosomal escape mechanisms to reach the cytosol and engage with target pathways [134]. The effectiveness of cargo release is influenced by the composition of vesicle membranes and the presence of lipids or proteins that facilitate fusion. Changes in these parameters affect the efficacy of vesicle cargo in modifying recipient cell activity.

A different technique for cargo transport is direct membrane fusion. In some instances, vesicle membranes amalgamate with the plasma membrane of the receiving cell, facilitating the direct transfer of their contents into the cytoplasm. This mechanism is contingent upon lipid composition, membrane fluidity, and the presence of fusogenic proteins [135]. Calcium levels and pH may influence fusion efficiency, hence connecting absorption processes to the physiological condition of the follicular environment. Vesicle function requires surface-bound signalling without total internalisation. Vesicle membrane proteins may attach to receptors on target cells, initiating intracellular signalling cascades [136]. PD-L1 on vesicles interacts with PD-1 on T cells, leading to inhibitory signalling via the recruitment of SHP-2 phosphatase and the suppression of downstream pathways. Alternative receptor–ligand combinations may facilitate such interactions. Vesicles may modify cellular behaviour without delivering their interior contents [137].

The outcome of these interactions is contingent upon the cell type. Granulosa cells internalise vesicles that regulate intracellular signalling pathways associated with metabolism, survival, and hormonal response. Immune cells recognise surface ligands and intracellular cargo, subsequently modifying their activation state and cytokine synthesis appropriately. The cumulus cells function as intermediaries, absorbing vesicles and transmitting signals to the oocyte via gap junctions or the release of secondary vesicles. The variations in receptor expression, membrane composition, and endocytic capability among various cell types dictate the relative distribution of vesicle-mediated signaling inside the follicle [138].

The timing and extent of signalling effects rely on intracellular trafficking subsequent to intake. Vesicles that evade degradation processes may sustain the control of gene expression via the continuous presence of microRNAs and proteins. Affecting receptor availability and signalling sensitivity may also be a consequence of the recycling of membrane components to the cell surface. Collectively, these mechanisms indicate that vesicle-mediated communication may coexist with cytokine signalling and direct cell–cell interactions, hence increasing the complexity of the follicular milieu.

### 7.5. Spatial Distribution and Functional Gradients Within the Follicle

Extracellular vesicles are unevenly distributed across the follicular cavity. Their localisation is dictated by fluid dynamics, cellular origin, and the physicochemical characteristics of the vesicle membrane. Follicular development and osmotic gradients generate slow convection currents in the antral fluid, leading to areas of varying vesicle concentration [139]. Vesicles released from mural granulosa cells enter the bulk fluid and migrate down these gradients, whereas cumulus cells generate vesicles that are contained within the extracellular matrix surrounding the oocyte. The cumulus matrix is abundant in hyaluronic acid, which enhances viscosity and restricts diffusion, efficiently sequestering vesicles in the pericellular space [38].

Vesicle mobility is contingent upon the composition of the membrane and its dimensions. Smaller vesicles with a lower lipid content diffuse more readily, while bigger vesicles or those with elevated cholesterol levels diffuse less readily [140]. The surface charge is significant since electrostatic interactions with matrix components or cell membranes may localise vesicles in certain regions. Moreover, interaction with extracellular matrix proteins like fibronectin and laminin further impedes diffusion and aids in the establishment of localised vesicle reservoirs. Vesicles are concentrated in microdomains that are significantly higher than the ambient fluid due to these factors [141].

The gradients generated by this distribution are associated with local variations in signalling intensity. Increased quantities of vesicles containing regulatory material, including checkpoint proteins and microRNAs, are seen in the regions surrounding the cumulus–oocyte complex [142]. In comparison to the more distant areas of the follicle, this creates a microenvironment where signalling inputs are amplified. Granulosa cells next to the basal lamina have a distinct vesicle profile, reflecting their closeness to the vascular supply and systemic signals. This spatial variability leads to region-specific regulation of cellular pathways [143].

Furthermore, temporal dynamics influence these gradients. The rates of vesicle release are modified throughout follicular development, particularly after hormonal stimulation and inflammatory signals linked to ovulation. Augmented secretion during the periovulatory stages leads to a temporary buildup in the follicular cavity [52]. Clearance mechanisms are present that reduce vesicle concentration over time. Some of these include the absorption by surrounding cells and the diffusion into the circulation following follicular rupture. The equilibrium distribution at each developmental stage is dictated by the balance between release and absorption [144].

Cellular barriers facilitate compartmentalisation. Physical restrictions, such as tight junctions and extracellular matrix structures inside the follicle, restrict vesicular mobility. Cumulus cells are interconnected by gap junctions within a dense network that restricts the entry of big vesicles while permitting the flow of tiny molecules [145]. Selective permeability regulates vesicle-mediated signalling adjacent to the oocyte via production and physical accessibility. Follicular variability stems from disparities in size, vascularization, and local cellular makeup. In bigger follicles with expanded antral gaps, flow patterns and vesicle distribution vary from those in smaller follicles [146]. Variations in cytokine concentrations and metabolic conditions affect vesicle generation and composition, resulting in distinct signalling environments even within a single ovary. The regional variations contribute to the variety of granulosa cell function and oocyte competence, suggesting a combination of spatial and molecular control. Given the central role of extracellular vesicles in follicular signaling, their main molecular components and functional effects are summarized below in Table 3.

## 8. Clinical Implications and Translational Perspectives

### 8.1. Follicular Fluid as a Molecular Window: Biomarkers and Stratification

The first layer comprises the cytokine profiles. The immunological environment is active, shown by interferon-γ, TNF-α, IL-6, and chemokines such CXCL10. Elevated levels do not inherently signify injury. They do suggest stress on the system. Checkpoint molecules provide contextual information. For instance, soluble PD-L1 indicates that regulation has been activated by that pressure [147]. Examining both concurrently provides a more comprehensive understanding than only assessing inflammation. A follicle that undergoes active cytokine production but maintains intact checkpoint signalling is distinct from one that lacks regulation [148].

Extracellular vesicles contain more precise information. Their composition mirrors the cells responsible for their secretion, namely granulosa cells and immune cells. Upon isolation, they may be measured and defined by dimensions, surface markings, and cargo. Vesicle-associated proteins and lipids indicate signalling activity, whereas their RNA content signifies continuing regulatory activities. The vesicles safeguard their contents, ensuring that the signal remains stable and more readily interpretable than if the molecules were dispersed in solution [149].

Patterns in steroidogenic genes, apoptotic pathways, or metabolic regulators differentiate growing and stressed follicles. Transcripts related to FSH responsiveness or mitochondrial activity often alter prior to the manifestation of any morphological alterations [150]. This makes them valuable as preliminary indications rather than as terminal markers. Consequently, by integrating these layers, each follicle may be more thoroughly described. One specimen exhibits immunological activity, regulatory equilibrium, vesicular signalling, and gene regulation. Variations across follicles within the same subject become evident. Certain follicles maintain a steady environment with balanced signalling. Some individuals seem agitated or dysregulated under microscopic examination, while they are not [151].

### 8.2. Immune Checkpoint Signaling as a Therapeutic Target

The follicular fluid offers insight into the oocyte’s immediate surroundings at this crucial stage. It is not a diluted systemic message. The local environment is regulated by granulosa cells, immune cells, and metabolic factors. Sampling during oocyte extraction accurately represents the environment without external intervention. Cytokines, checkpoint proteins, vesicles, and short RNAs coexist in the same environment, each embodying a distinct aspect of follicular activity [152,153,154].

MicroRNAs inside these vesicles provide insight into the regulatory mechanisms governing granulosa cell activity. Patterns in steroidogenic genes, apoptotic pathways, or metabolic regulators differentiate growing and stressed follicles. Transcripts related to FSH responsiveness or mitochondrial activity often alter prior to the manifestation of any morphological alterations. This makes them valuable as preliminary indications rather than as terminal markers [155]. By integrating these layers, each follicle may be more thoroughly described. One specimen exhibits immunological activity, regulatory equilibrium, vesicular signalling, and gene regulation. Variations across follicles within the same subject become evident. Certain follicles maintain a steady environment with balanced signalling. Some individuals seem agitated or dysregulated under microscopic examination, while they are not. This facilitates an alternative approach to oocyte selection. Molecular information may be used earlier in the process, rather than only for post-fertilization morphology. Increased oocyte maturation is more probable in follicles with a more stable signalling profile. Individuals with disturbed patterns continued to generate oocytes but with diminished developmental potential [156].

### 8.3. Extracellular Vesicles as Diagnostic and Therapeutic Tools 

Extracellular vesicles contain more precise information. Their composition mirrors the cells responsible for their secretion, namely granulosa cells and immune cells. Upon isolation, they may be measured and defined by dimensions, surface markings, and cargo. Vesicle-associated proteins and lipids indicate signalling activity, whereas their RNA content signifies continuing regulatory activities. Because the vesicles safeguard the contents within, the signal is more stable and simpler to interpret than if the molecules were free in solution [157,158].

MicroRNAs inside these vesicles provide insight into the regulatory mechanisms governing granulosa cell activity. Patterns in steroidogenic genes, apoptotic pathways, or metabolic regulators differentiate growing and stressed follicles. Transcripts related to FSH responsiveness or mitochondrial activity often alter prior to the manifestation of any morphological alterations. This makes them valuable as preliminary indications rather than as terminal markers. By integrating these layers, each follicle may be more thoroughly described [159]. One specimen exhibits immunological activity, regulatory equilibrium, vesicular signalling, and gene regulation. Variations across follicles within the same subject become evident. Certain follicles maintain a steady environment with balanced signalling. Some individuals seem agitated or dysregulated under microscopic examination, while they are not.

### 8.4. Integration into Assisted Reproduction Strategies

The translation of these molecular insights into the clinic requires little disruption to existing workflows. Follicular fluid is already collected during oocyte retrieval, so no other invasive steps are necessary. The difference is in how you utilise this material. It is not a waste but a source of information on the follicular environment at the time of oocyte collection [160]. Molecular profiling can help inform decisions at multiple points in the IVF process. Information on immune activity, vesicle content and signalling balance may assist in identifying which oocytes are developing in favourable conditions. The following may impact decisions on the timing of embryo transfer, or whether to transfer fresh or cryopreserve. In cycles with heterogeneous follicular profiles, it may also account for differences in embryo quality not predicted by morphology alone [148].

With molecular data and clinical parameters, more precise stratification of patients is possible. Age, BMI and ovarian reserve markers provide a general framework but do not capture the local follicular conditions. Adding cytokines information, checkpoint signalling and vesicle profiles adds a layer of real-time biology [161]. The following may help differentiate between patients who are clinically similar but molecularly different. Once these differences are identified, specific interventions become possible. Interventions to reduce immune activation might be useful in patients with persistent inflammation. Others with impaired metabolic signalling may require modifications of stimulation protocols or supportive treatments. The aim is not to deliver one intervention, but to adjust the intervention to the current signalling pattern in each patient [162].

Predictive models can improve, too. Current models are based mainly on demographic and hormonal data. The accuracy of these markers may be increased by adding follicular fluid molecular markers. Patterns are likely to be more useful than single values, especially in the case of complex systems such as the follicle. Machine learning approaches may help identify combinations of markers that correlate with outcomes, but this requires large, well-characterised datasets. Routine implementation must follow validation. Results require replication in other populations and clinical settings. Decision-making must be guided by standard thresholds. Without this, molecular data are at risk of being descriptive rather than actionable. However, if validated, follicular fluid analysis might become a useful tool to support more individualised and biologically informed IVF strategies.

## 9. Discussion

### 9.1. Extracellular Vesicles and Immune-Regulatory Signaling in the Follicular Microenvironment

Johnson et al. characterise PD-1/PD-L1 as an active regulatory system situated intra-follicularly, with expression in granulosa cells, oocytes, and follicular fluid, including both soluble and exosomal forms that may suppress IFN-γ production [7]. In the context of Guleria et al., who demonstrated that PD-L1 deficiency results in loss of fetomaternal tolerance via uncontrolled Th1 responses, and Zeng et al., who demonstrated that PD-L1 blockade results in altered Tfr dynamics and increased foetal resorption, this observation is even more significant mechanistically [163,164]. In all these models, a singular axis governs the equilibrium between effector and regulatory immunity. Within the follicle, PD-L1 not only inhibits immune activity but also determines the quality of the immune response, obstructing a transition to IFN-γ-dominated signalling that might potentially undermine granulosa cell function.

Knapik et al. complement this view by characterising the ovary as an immunologically active organ, whereby T lymphocytes play roles in both tolerance and disease [165]. Research aligns with researchers who noted the physiological presence of immune cells in ovarian tissue, and with Sedmak et al., who showed that the absence of immunological control leads to autoimmune oophoritis [166,167]. Kobayashi et al. demonstrated that Treg dysfunction leads to ovarian failure, highlighting the significance of controlled immune signalling in preserving follicular integrity [168]. Collectively, these results indicate a regulated immunological balance inside the follicle, where regulatory mechanisms, such as PD-L1 signalling, are essential to avert the onset of pathogenic inflammation.

Bortot et al. and Johnson et al. concur on the function of extracellular vesicles as transporters of PD-L1 in follicular fluid [7,8]. Selective packaging, rather than inert release, is indicated by the identification of PD-L1+ vesicle subpopulations, notably those associated with CD9. This method facilitates checkpoint signalling at a distance from direct cell-to-cell interaction. PD-L1 may engage with T cells in the follicular cavity and transmit regulatory cargo to granulosa cells via vesicle-mediated transport. Integration with the results of Albeitawi et al., who characterise follicular fluid as a multifaceted signalling medium including many classes of biomarkers, indicates that vesicle-associated PD-L1 is integral to a more extensive molecular network orchestrating follicular communication [169].

Han et al. establish a clear correlation between PD-L1 and intracellular signalling, illustrating the activation of PI3K/AKT and a reduction in granulosa cell death [9]. Such findings are especially pertinent when considered alongside Jiao et al., who showed that IFN-γ and TNF-α provoke granulosa cell death and impede steroidogenesis via the regulation of CYP19A1. Conversations of these pathways suggest that PD-L1 not only suppresses inflammatory signals but also modifies their downstream effects. The preservation of AKT activity stabilises mitochondrial function and limits caspase activation, allowing granulosa cells to survive amid pro-apoptotic signals.

Zhong et al. expand this intracellular aspect by illustrating the role of post-translational changes in regulating granulosa cell proliferation, differentiation, and death [170]. PD-L1 also affects pathways, including phosphorylation-dependent signalling pathways, which are subject to these modifications. Glycosylation, ubiquitination, and acetylation may modulate the stability and functionality of proteins, offering an additional mechanism via which checkpoint signals might indirectly influence cellular outcomes. Consequently, it has been shown that PD-L1 stability is likewise governed by analogous post-translational processes, connecting checkpoint expression with intracellular regulatory systems.

Liu et al. and Banerjee et al. elucidate immunological and cytokine alterations in obesity and PCOS, including heightened infiltration of CD4+ T cells, elevated levels of IL-4, IL-6, and TNF-α, as well as enduring inflammatory markers in follicular fluid [23,121]. STAT and NF-κB pathway activation in granulosa cells is sustained by these environments. Under these conditions, PD-L1 expression is presumably perpetually stimulated by interferon signalling as a compensatory mechanism. However, prolonged activation of survival pathways does not fully restore functional capability, as shown by Han et al. Granulosa cells maintain viability but exhibit compromised steroidogenesis and modified metabolic signalling, suggesting a transition to a survival-oriented phenotype [9].

Poor ovarian response is further exacerbated by Zhou et al., who demonstrate its association with changed immune cell composition, including an increase in dendritic cells and modifications in macrophage polarization [171]. Accelerated antigen presentation and cytokine secretion, which contribute to sustaining a continuous signalling milieu. Continual activation of interferon-related pathways leads to an increased dependence on checkpoint control to avert excessive harm. Prolonged activation of PD-1 may hinder signalling flexibility, therefore restricting the capacity of granulosa cells to adaptively react to hormonal signals. Han et al. demonstrate that cytokine dysregulation (increased IP-10, modified CD4+/CD8+ ratios) directly affects gene expression in granulosa cells, resulting in decreased levels of FSHR and CYP19A1 [9]. This is a straightforward instance of immunological signalling resulting in endocrine dysfunction. But when considered in the context of PD-L1 modulation of interferon signalling, a pattern arises in which checkpoint activity attempts to mitigate these effects; however, it is unable to completely reverse them in the context of persistent inflammation. Piccinni et al. demonstrate that cytokine profiles in follicular fluid are meticulously maintained and affect follicle formation independently of systemic gonadotropins, even during physiological cycles [172]. Immune signalling is an intrinsic component of folliculogenesis, as indicated by this information, rather than a pathological addition.

Jiao et al. elucidate a strong molecular basis, demonstrating that Th1 dominance via IFN-γ and TNF-α directly promotes granulosa cell death and disrupts steroidogenesis by downregulating CYP19A1 and CTGF [173]. Han et al. (endometriosis) observed elevated IP-10 levels and modified CD4^+^/CD8^+^ ratios in follicular fluid, along with direct inhibition of FSHR and CYP19A1 expression in granulosa cells after cytokine exposure [9]. Collectively, our data indicate a coherent mechanism by which interferon-mediated signalling transitions granulosa cells from a steroidogenic phenotype to a stress–response state. Activation of STAT1 and NF-κB, as well as transcriptional programs that promote apoptosis, inflammation, and decreased endocrine output, are observed in conjunction with this alteration. Liu et al. expand this paradigm to obesity, demonstrating heightened CD4^+^ T-cell infiltration and elevated levels of IL-4 and IL-13 in the follicular milieu [121]. Banerjee et al. observed the same results in PCOS, indicating increased cytokines and changed immune-cell composition even post-ovarian stimulation [174]. The granulosa cells are believed to be subjected to a consistent cytokine signalling, as opposed to a transient immune activation, according to these studies. Prolonged exposure leads to persistent activation of STAT3 and NF-κB, which interferes with normal signalling via FSHR and cAMP-PKA pathways. Phosphorylation of CREB is diminished, resulting in a decrease in the transcription of steroidogenic enzymes. Metabolic processes transition from biosynthesis to stress adaptation.

Zhou et al. provide the aspect of immune-cell architecture in inadequate ovarian response, revealing a decrease in total macrophages with an elevation in polarisation signals and an expansion of dendritic cell subsets [171]. This pattern indicates instability rather than an absence of competence. Modifications in macrophage signalling influence the release of cytokines and growth factors that govern extracellular matrix remodelling and angiogenesis. Simultaneously, dendritic cell-mediated antigen presentation sustains T-cell activation, hence preserving follicular interferon signalling. The collective activity of these cells creates a milieu whereby granulosa cells are perpetually exposed to pro-inflammatory stimuli without resolution.

Knapik et al. assert that immune cells are not aberrant components but rather integral actors in ovarian function [165]. The disruption of immunological control leads to autoimmune oophoritis, as indicated by Sedmak et al. Kobayashi et al. show that Treg dysfunction contributes to ovarian failure [166,168]. The research characterised immunological signalling as a continuum rather than a dichotomous system. Granulosa cells exhibit varying responsiveness to the equilibrium of regulatory and effector signals within this range. Excessive Th1 activity results in apoptosis and functional deterioration, whereas uncontrolled activity permits the advancement of inflammatory damage.

Zhong et al. elucidate a molecular bridge that regulates the signalling pathways of granulosa cells via post-translational alterations [170]. Phosphorylation, acetylation, and ubiquitination regulate essential proteins implicated in proliferation, apoptosis, and steroidogenesis. Such modifications intersect with the cytokine-driven pathways described by Jiao et al. and Han et al., which either enhance or repress signalling outputs depending on the intracellular context [9,173]. Changes in protein stability and enzyme activity also influence granulosa cell responses, linking external immunological signalling to intracellular control. Brązert et al. complicate the scenario by revealing substantial transcriptional reprogramming of granulosa cells in vitro, characterised by altered expression of genes linked to mitotic spindle construction and cell cycle control [175]. This research, although concentrating on culture conditions, indicates that granulosa cells have heightened sensitivity to environmental cues. From the standpoint of inflammatory research, this indicates that cytokine exposure in vivo might potentially elicit analogous alterations in cell-cycle dynamics and structural organization, affecting both proliferation and differentiation.

Albeitawi et al. describe follicular fluid as a complex signalling environment including many kinds of biomarkers, including cytokines, hormones, and metabolic variables [169]. In conjunction with disease-specific data, this viewpoint indicates that granulosa cell activity is regulated by a network of interrelated signals rather than by discrete pathways. Cytokines alter hormone synthesis, metabolic alterations influence signalling pathways, and immune cells concurrently regulate both processes. The following interconnected system elucidates how alterations in a single component are conveyed across the whole follicular milieu.

### 9.2. Disease-Associated Disruption of Follicular Immune and Metabolic Regulation

Albeitawi et al. view follicular fluid as a dense, interacting molecular system, whereas Johnson et al. and Bortot et al. see PD-L1 in extracellular vesicles as an active signalling unit instead of a byproduct of membrane recycling [7,8,169]. When united, vesicles serve as the structural underpinning that organises this complexity. Bortot et al. revealed PD-L1 enrichment in some vesicle subpopulations, such as CD9-associated vesicles, which shows that cytokine-activated intracellular pathways likely underlie selective trafficking [8]. Liu et al. and Banerjee et al. demonstrate sustained cytokine activity in PCOS and obesity, particularly regarding IL-6, TNF-α, and IL-4 [121,174]. These cytokines trigger STAT and NF-κB signalling, which control vesicle biogenesis machinery and RNA-binding proteins. Thus, the inflammatory pathways outlined in these investigations influence granulosa cell transcription and determine the molecules exported by vesicles, establishing a clear connection between intracellular immune signalling and external communication.

Han et al. (PCOS) and Jiao et al. (granulosa cells) elucidate persistent IFN-γ and TNF-α stress resulting in apoptosis and steroidogenic dysfunction via the inhibition of CYP19A1 and the disruption of survival pathways [9,173]. Johnson et al. reveal that follicular fluid includes vesicles abundant in PD-L1 that may inhibit IFN-γ production, whereas Bortot et al. identify particular vesicle subtypes that may convey this checkpoint signal [7,8]. When examined together, a feedback loop becomes evident. Cytokine exposure stimulates PD-L1 expression and vesicle packing, whereas vesicle-released PD-L1 subsequently inhibits T-cell activation and IFN-γ production. This interaction does not eliminate inflammatory signals but adjusts their intensity. The concurrent transport of microRNAs in vesicles that target the same pathways as cytokines enhances the suppression of steroidogenic genes at many regulatory tiers. Zhong et al. elucidate that post-translational alterations govern essential signalling proteins, suggesting that vesicle cargo may indirectly influence the phosphorylation and stability of these proteins, hence amplifying the downstream effects of cytokine signalling [170].

Zhou et al. found alterations in immune-cell architecture in cases with inadequate ovarian response, characterised by an increase in dendritic cells and unstable macrophage polarization [171]. Knapik et al. and Abdulrahman et al. both emphasise that immune cells are essential to ovarian function rather than exogenous disruptors [165,167]. Collectively, these data indicate that vesicles originating from immune cells are always engaging with vesicles produced by granulosa cells inside the follicle. Dendritic cell-derived vesicles may convey antigen-presenting molecules and cytokine signals that sustain T-cell activation, whereas macrophage-derived vesicles may transmit inflammatory mediators and metabolic regulators. A multi-source signalling network is established by the merger of these inputs with granulosa cell vesicles. Sedmak et al. and Kobayashi et al. illustrate that compromised immunological control leads to ovarian dysfunction, reinforcing the notion that disruption of this vesicle-mediated communication pathway might precipitate pathological conditions [166,168].

Han et al. demonstrate that cytokines, including IP-10, directly suppress the expression of FSHR and CYP19A1 [9]. Piccinni et al. demonstrate that cytokine profiles influence follicular growth independently of systemic hormones, even under physiological settings [172]. When these results are synthesised, vesicles may be regarded as carriers that stabilise and transmit the effects of these cytokines. The persistence of pathway regulation is facilitated by vesicle-delivered microRNAs and proteins rather than by transitory signalling. Moreover, Banerjee et al. show that immune profiles change between systemic circulation and follicular fluid, indicating localised modulation [174]. Vesicles serve as a strategy for preserving compartmentalisation, enabling follicle-specific signalling settings to function independently of systemic circumstances.

Johnson et al. and Bortot et al. expand this notion into the follicle via PD-L1-containing vesicles [7,8]. Vesicle-mediated checkpoint delivery resembles a pre-implantation tolerance mechanism. PD-L1 contained in vesicles may suppress IFN-γ production, whereas microRNA cargo may modulate other immune pathways, thereby creating a decentralised tolerance network inside the follicular cavity. Stable cell–cell connections are not necessary for this system to function, which enables precise local regulation of immune responses.

Brązert et al. illustrate that the gene expression of granulosa cells is significantly influenced by environmental factors, including alterations in cell-cycle and structural genes [175]. Zhong et al. demonstrate that these alterations are facilitated by post-translational modifications and the modulation of signalling pathways [170]. The recurrent internalisation of vesicles with analogous regulatory cargo, in conjunction with vesicle biology, offers a method for maintaining these transcriptional alterations. Ongoing exposure to vesicle-mediated signals may enhance or inhibit certain pathways, leading to persistent phenotypic alterations in granulosa cells.

Han et al. contextualised PCOS by explicitly associating PD-L1 signalling, granulosa cell death, and PI3K/AKT activity [9]. When considered with the research of Banerjee et al. and Liu et al., which demonstrate that PCOS and obesity are both systemic inflammatory illnesses and follicle-specific immunological disorders, their results get further validation [121,174]. Banerjee et al. delineate distinctive cytokine and immune cell profiles in the follicular fluid of individuals with PCOS [174]. Liu et al. demonstrate an elevated presence of T-cells and modified cytokine transcription in overweight and obese women [176]. Collectively, these investigations indicate ongoing signalling to granulosa cells in metabolically compromised follicles via STAT, NF-κB, insulin receptor, and PI3K-related pathways. PD-L1 overexpression may represent a compensatory response to persistent inflammatory signaling that is insufficient to fully restore follicular balance. Nevertheless, the precise contribution of checkpoint communication to different reproductive disorders remains poorly defined.

Han et al. provide an alternative viewpoint on the subject of endometriosis [9]. Their research indicated a decreased CD4+/CD8+ ratio, elevated levels of IP-10, RANTES, and G-CSF, along with direct suppression of FSHR and CYP19A1 in granulosa cells. This, together with Jiao et al.’s evidence that IFN-γ and TNF-α induce granulosa cell death and steroidogenic failure, elucidates a shared inflammatory axis. Consequently, endometriosis may hinder follicular competence via chemokine-mediated T-cell recruitment and by directly interfering with gonadotropin response. Johnson et al. and Bortot et al. introduce the checkpoint dimension: PD-L1 in follicular fluid and vesicles may seek to mitigate this inflammatory response [7,8]. Most of the available evidence is still derived from observational studies or experimental models, whereas direct functional data from human follicles remain limited.

Zhou et al. demonstrate that inadequate ovarian response has a distinct immunological profile characterised by altered macrophage polarisation, elevated dendritic cell subsets, and changed T-cell distribution [171]. This profile, albeit different from those of PCOS and endometriosis, nonetheless aligns with the same downstream targets: granulosa cell signalling, steroidogenesis, and oocyte maintenance. Knapik et al. elucidate this discovery by demonstrating that ovarian immune cells are a component of normal physiology [165]. Sedmak et al. and Kobayashi et al. illustrate the consequences of regulatory failure, resulting in ovarian tissue progressing towards immune-mediated dysfunction [166,168]. Excessive inflammation may not be the only issue with POR. Disordered immune architecture may result in unstable signalling, whereby dendritic cells, macrophages, and T cells provide inadequately coordinated inputs to granulosa cells.

Piccinni et al. present a significant physiological difference [172]. Cytokine and hormone levels fluctuate with follicle state and age; however, control remains coordinated. Albeitawi et al. expand this to IVF/ICSI, demonstrating that follicular fluid indicators interact via hormonal, inflammatory, oxidative, metabolic, and microRNA pathways [169]. Pathology does not generate a new follicular system, according to these physiological and clinical biomarker investigations compared to illness-specific data. It transforms a pre-existing entity. The cytokines typically associated with follicular development become excessive or misaligned. Typically, vesicles facilitate cellular communication; nevertheless, they are now beginning to carry stress-related materials. Checkpoint circuits that usually restrict immune activation may transform into regulatory mechanisms instead of compensatory ones.

### 9.3. Clinical Translation: From Follicular Immune Profiling to IVF Decision-Making

PD-L1 is detectable in follicular fluid in both soluble and vesicle-bound forms, and follicular fluid is regarded as a multi-layered biomarker reservoir that reflects oocyte competence and IVF results [7,8,169]. In conjunction with Piccinni et al., this has a clear translational implication, as they have shown that follicular development is already influenced by cytokine and hormone concentrations during physiological cycles [172]. Follicular fluid is not only a byproduct of retrieval. It is a physiologically active matrix that encodes the immunological, endocrine, and metabolic state of each follicle. A direct molecular readout of the oocyte microenvironment that is not reflected by systemic markers may be obtained by measuring checkpoint proteins, cytokines, vesicle cargo, and metabolic markers at the time of oocyte retrieval.

Banerjee et al. and Liu et al. demonstrate that immunological profiles vary between circulation and follicular fluid in PCOS and obesity, demonstrating that systemic inflammation does not entirely represent the follicular condition [174,176]. Han et al. linked these inflammatory circumstances to intracellular pathways, revealing PD-L1-mediated stimulation of the PI3K/AKT pathway and decreased apoptosis in granulosa cells [9]. Jiao et al. demonstrate that IFN-γ and TNF-α concurrently influence steroidogenesis impairment and the activation of apoptosis [173]. The data together suggest that follicles under metabolic stress may seem alive because of the preservation of survival signals; however, they are functionally impaired. This has direct consequences for in vitro fertilisation. Oocytes from these follicles may undergo fertilization. But they possess genetic abnormalities that impair embryo development, which cannot be identified by appearance alone.

Han et al. and Zhou et al. provide disease-specific immune markers that may facilitate classification [9,171]. Han et al. correlated IP-10, RANTES, and modified CD4+/CD8+ ratios with decreased fertilisation and live birth rates [9]. Zhou et al. discovered that dendritic cells, macrophages, and CD4+ T cells are indicative of a suboptimal ovarian response. Both Knapik et al. and Abdulrahman agree that immune cells play an important role in ovarian physiology, and their findings are in line with ours [167,171]. Ovarian pathology resulting from the disturbance of immunological control [166,168]. A paradigm in which each clinical condition is associated with a distinct follicle immune architecture is collectively supported by these studies. Clinical translation should go towards immunological phenotyping of follicles instead of using a standardised inflammatory marker for all individuals.

Johnson et al., Bortot et al., and Zhong et al. include extracellular vesicles and post-translational regulation into this clinical context. Vesicle-associated PD-L1 and microRNA cargo indicate intracellular signalling states [7,8,170]. Zhong et al. illustrate that granulosa cell activity is governed by phosphorylation, ubiquitination, and other changes [170]. Brązert et al. demonstrate the impact of environmental alterations on granulosa cell gene expression [175]. The findings suggest that vesicle profiling may indirectly provide insights into the activation of intracellular circuits. The assessment of vesicle cargo may suggest the activation of PI3K/AKT, NF-κB, or stress pathways without requiring intrusive cellular examination. In contrast, several relationships are now based on indirect vesicle profiling studies and need adequate validation in vivo within the human follicular milieu.

Recent computational studies indicate that the control of PD-L1 may also rely on dynamic conformational processes. Li et al. conducted molecular dynamics simulations that revealed small-molecule-mediated stabilization of a closed PD-L1 dimer configuration could disrupt the PD-1/PD-L1 interaction interface, with several hotspot residues identified as potential determinants of checkpoint stability and signaling behaviour [177]. Despite being derived from a non-reproductive context, our data underscore the intricate structural and regulatory nature of PD-L1-associated signaling pathways.

The existing literature has several significant shortcomings. It remains uncertain whether PD-1/PD-L1 transmission actively governs follicular immune homeostasis in vivo or only indicates local inflammatory activity. The functional relevance of PD-L1-positive extracellular vesicles in human follicular fluid has yet to be explicitly verified. Third, the majority of the existing information derives from observational research, in vitro tests, or extrapolations from other immune-regulatory systems, rather than direct mechanistic investigations in humans. Fourth, there exists considerable methodological variability in follicular fluid collection, extracellular vesicle separation, and molecular characterization techniques. The paucity of defined criteria for immune-checkpoint-related indicators and the lack of prospective validation studies are now hindering the practical use of these markers in IVF treatment.

Initially, it must be ascertained if PD-1/PD-L1 communication actively regulates follicular immunological equilibrium in vivo or whether its presence is mostly a result of local inflammatory activation. Future studies should investigate whether heightened follicular PD-L1 expression signifies follicles engaged in active compensatory immune regulation rather than just reflecting increased inflammation. The functional validation of PD-L1 associated with extracellular vesicles in facilitating immune regulation in human follicular fluid has yet to be conducted. This should be investigated by isolating PD-L1-positive vesicle subpopulations and evaluating their influence on T-cell activation, granulosa cell signalling, and oocyte-related outcomes. Third, biomarker studies are constrained by varying sampling methodologies, heterogeneous IVF cohorts, and the absence of standardised clinical criteria. Future study should assess if a composite follicular-fluid profile of cytokines, checkpoint proteins, and vesicle cargo serves as a superior predictor of oocyte competence compared to traditional clinical and embryological indicators.

## 10. Conclusions and Future Directions

The ovarian follicle is a dynamic environment where inflammatory indicating, immunological regulation, granulosa cell function, and metabolic activity interact during oocyte maturation. Current evidence suggests that PD-1/PD-L1 signaling may participate in maintaining follicular homeostasis under both physiological and pathological conditions. Alterations in the inflammatory and immunological characteristics of the follicular compartment have consistently been linked to conditions such as PCOS, endometriosis, obesity, and suboptimal ovarian response. Extracellular vesicles and cytokine signaling may also contribute to the local immunological and metabolic control concurrently. Nevertheless, many processes now suggested are mostly supported by indirect evidence obtained from observational research, in vitro systems, and translational extrapolation from other immune-regulatory settings. Follicular immune-checkpoint signaling is emerging as a potentially relevant area of reproductive and translational research. Consequently, more mechanistic and translational investigations are necessary to clarify the specific function of immune modulation within the follicular milieu and its possible ramifications for assisted reproduction.

## Figures and Tables

**Table 1:** Immune cell populations and their functional roles in the follicular microenvironment.

Cell Type	Key Mediators	Molecular Pathways	Functional Role in Follicle	Clinical Relevance
CD4^+^ T cells	IL-4, IL-6, IFN-γ	JAK/STAT, NF-κB	Regulation of inflammatory tone	Altered in PCOS, obesity
CD8^+^ T cells	IFN-γ, TNF-α	STAT1, apoptosis pathways	Cytotoxic signaling, granulosa stress	Increased in endometriosis
Treg cells	IL-10, TGF-β	FOXP3 signaling	Immune tolerance, suppression of Th1	Reduced in ovarian failure
Macrophages (M1/M2)	TNF-α, IL-1β, growth factors	NF-κB, MAPK	Tissue remodeling, inflammation control	Altered in POR
Dendritic cells (cDC1/cDC2)	IL-12, antigen presentation	T-cell activation pathways	Immune activation and regulation	Predictor of POR
Granulosa cells (immune-active)	PD-L1, cytokines	PI3K/AKT, NF-κB	Immune modulation, oocyte support	Dysfunction in all pathologies

**Table 2:** Disease-specific immune and molecular alterations in the follicular microenvironment.

Condition	Immune Profile	Key Cytokines	Intracellular Pathways	Granulosa Cell Effect	IVF Impact
PCOS	Chronic low-grade inflammation	IL-6, TNF-α, IL-4	PI3K/AKT, STAT3	Reduced steroidogenesis, survival maintained	Variable embryo quality
Obesity	Metabolic inflammation	IL-6, IL-1β, TNF-α	NF-κB, AMPK dysregulation	Mitochondrial dysfunction	Lower embryo quality
Endometriosis	Th1-dominant, chemokine-driven	IP-10, RANTES, G-CSF	STAT1, NF-κB	↓ FSHR, ↓ CYP19A1	Reduced fertilization & LBR
POR	Immune instability	Mixed cytokine profile	Impaired PI3K/AKT	Reduced metabolic support	Low oocyte yield
Physiological	Balanced immune tone	Controlled cytokine levels	Integrated signaling	Normal function	Optimal outcomes

**Table 3:** Extracellular vesicle cargo and functional impact in the follicular environment.

EV Component	Molecular Target	Pathway Affected	Functional Effect	Clinical Implication
PD-L1	PD-1 receptor	SHP2, PI3K inhibition	T-cell suppression	Immune tolerance
miRNAs (e.g., targeting CYP19A1)	mRNA degradation	Steroidogenesis pathways	↓ Estrogen synthesis	Poor oocyte quality
miRNAs (PI3K/AKT-related)	AKT signaling	Survival pathways	Altered apoptosis balance	PCOS relevance
Proteins (kinases/phosphatases)	Signaling cascades	NF-κB, MAPK	Signal amplification	Chronic inflammation
Metabolic enzymes	Cellular metabolism	Glycolysis, ROS	Energy imbalance	Embryo competence
Lipids	Membrane signaling	Lipid raft signaling	Receptor modulation	Metabolic disorders

## Data Availability

No new data were created or analyzed in this study. Data sharing is not applicable to this article.
